# A Review of Fluoroquinolones with a Focus on Veterinary-Approved Agents

**DOI:** 10.3390/biom16070984

**Published:** 2026-07-03

**Authors:** Joseph M. Blondeau

**Affiliations:** 1 Clinical Microbiology, Royal University Hospital and the Saskatchewan Health Authority, Saskatoon, SK S7N 0W8, Canada; joseph.blondeau@saskhealthauthority.ca; 2 Pathology and Laboratory Medicine and Ophthalmology, Departments of Biochemistry, Microbiology and Immunology, University of Saskatchewan, Saskatoon, SK S7N 0W8, Canada

**Keywords:** fluoroquinolones, veterinary medicine, antimicrobials

## Abstract

Fluoroquinolones are broad-spectrum, bactericidal antibacterial agents used in both human and veterinary medicine. Some human-approved drugs are used off label in veterinary medicine while veterinary-approved drugs are not used in humans. Veterinary-approved fluoroquinolones are used on both food and companion animals and against a wide range of clinical indications including infections of the urinary tract, respiratory tract, skin and skin structure, mammary gland and others. Not all veterinary-approved fluoroquinolones have the same clinical indication and it is important to understand these important differences. A recently approved for food animals agent called pradofloxacin is characterized as being dual targeting in that it simultaneously inhibits DNA gyrase (topoisomerase II) and Topoisomerase IV—two enzymes critical for DNA replication. Simultaneous targeting of two enzymes is argued to reduce the likelihood for resistance selection. This article reviews veterinary-approved fluoroquinolones including an overview of the drugs, in vitro activity including bactericidal properties, pharmacokinetic/pharmacodynamics, antimicrobial resistance, anti-inflammatory properties and clinical trial results. Appropriate use of this important class of antimicrobial agents is essential for clinical success and long-term viability of these compounds.

## 1. Introduction

Fluoroquinolones are bactericidal broad-spectrum antibacterial agents that are used in both human and veterinary medicine. In human medicine, fluoroquinolones are classified as critically important by the World Health Organization. In veterinary medicine, fluoroquinolones are considered critical drugs due to their potency, excellent tissue penetration and life-saving therapy in food and companion animals. Broadly, these compounds have in vitro and clinical activity against Gram-negative and Gram-positive bacteria, atypical pathogens such as *Chlamydia*, *Mycoplasma* and *Mycobacterium* species and depending on the specific fluoroquinolone agent (e.g., moxifloxacin human, pradofloxacin veterinary), activity against anaerobic bacteria. The clinical indications for each fluoroquinolone varies, being broadest for ciprofloxacin and levofloxacin in human medicine and more restrictive for drugs such as norfloxacin due to poor systemic distribution. In veterinary medicine enrofloxacin has broad clinical use in companion, food and exotic animals [1,2]. Veterinary-approved fluoroquinolones are not used in human medicine; however, some human-approved agents are used in veterinary medicine—even though they are not approved for the clinical indications in which they are used [3,4].

Fluoroquinolones, like other drug classes, are not all the same and understanding the distinct difference between compounds is necessary for their correct and optimal use. This review article is intended as a comprehensive narrative review of fluoroquinolone agents focusing on veterinary-approved drugs with some data also related to human-use-approved agents where insufficient data exists for veterinary-approved drugs. The review is intended to capture the extensive literature and broad scope of data on fluoroquinolones in veterinary medicine.

Literature searches were conducted for fluoroquinolones and for each individual fluoroquinolone agent. Summaries were written for each heading/subheading (i.e., mechanisms of action, pharmacology, clinical indications, etc.) considering the published reports and the authors expertise on this class of drugs. The review article was constructed from this approach. The manuscript was written entirely by the author and no forms of artificial intelligence were used for any aspect of the manuscript.

**Figure 1:**
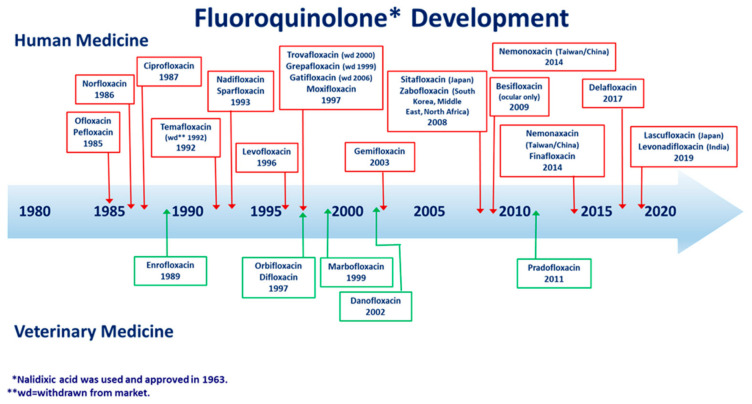
A chronology of the development of fluoroquinolones in human and veterinary medicine.

Figure 1 shows a chronology of fluoroquinolones in human and veterinary medicine. Table 1 and Table 2 summarize veterinary-approved fluoroquinolones in food and companion animals. The approved organisms on the label and clinical indications are also included.

## 2. Fluoroquinolone Classification and Chemistry

**Figure 2:**
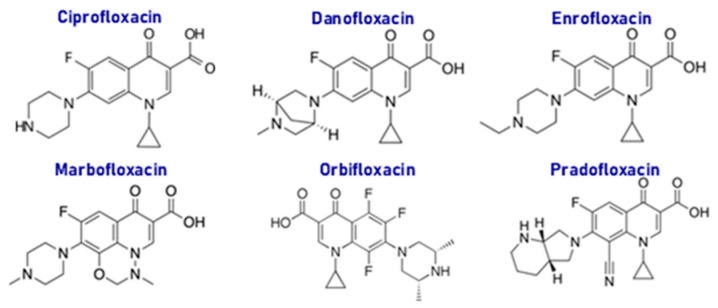
Clinical structures of veterinary fluoroquinolones and ciprofloxacin.

Fluoroquinolones developed for use in both human and veterinary medicine have been divided into generations. Figure 2 shows the chemical structures of select veterinary-approved fluoroquinolones. In general, the differentiation into various generations is based on alterations to the chemical structure, spectrum of activity, and pharmacokinetic/pharmacodynamic properties. First generation quinolones included nalidixic acid [8] and cinoxacin [9], but their utility was limited to treatment of urinary tract infections in humans. The addition of a fluorine at position C6 and a cyclopropyl group at position R1 of the quinolone molecule gave rise to fluoroquinolones and second-generation agents. The second-generation agents had overall enhanced activity and specifically enhanced activity against Gram-negative bacteria. Second-generation agents included ciprofloxacin, enoxacin, norfloxacin, ofloxacin and lomefloxacin. The addition of a chloro group at position R8, which enhances molecule activity against Gram positive and atypical bacteria gave rise to third generation agents which included fleroxacin, levofloxacin, sparfloxacin, grepafloxacin and gatifloxacin. Further molecule modifications included a cyclic diamine piperazine molecule at position C-7 and a methoxy group at the C-8 position. Fourth generation agents included gemifloxacin (no longer marketed), moxifloxacin and trovafloxacin. Various adverse effects resulted in the withdrawal of some of the quinolone agents from the clinical market such as grepafloxacin and trovafloxacin and the drug gatifloxacin was withdrawn from systemic use in humans but remains as the active ingredient in ophthalmic formulations.

In veterinary medicine, enrofloxacin, difloxacin and marbofloxacin are considered second-generation agents, danofloxacin and orbifloxacin are third generation agents and pradofloxacin is a fourth generation agent. Others have suggested a slightly different stratification of generations with pradofloxacin as a third generation agent [10]. Specifically, for pradofloxacin, a pyrrolidine group at position seven of the quinolone ring provides a broader spectrum of activity and has a lower propensity for resistance selection. Additionally, pradofloxacin (like moxifloxacin in humans) simultaneously targets DNA gyrase (topoisomerase II) and topoisomerase IV—enzymes critical for bacterial DNA replication—and as a dual targeting drug has a reduced likelihood for resistance selection (discussed in detail later).

## 3. Mechanism of Action/Mechanism of Resistance

Antimicrobial agents selectively inhibit critical functions in bacterial cells giving rise to either a bactericidal or bacteriostatic effect [11]. For the bacteriostatic effect, inhibition of ribosomal function or a metabolic pathway prevents bacterial growth and inhibition but does not result in bacterial death, as regrowth would occur if the antibiotic was removed or when antibiotic concentrations drop below the value necessary to inhibit the bacterial function. In contrast, bactericidal agents are lethal to the organism and irreversibly block or interfere with a function or structure essential for organism survival. For example, beta lactam agents (penicillin, cephalosporins, carbapenems, monobactams) bind to penicillin-binding proteins (PBPs)—a group of proteins called transpeptidases that are essential for formation of peptidoglycan, which we know is a critical cell wall structure necessary for bacteria survival [12]. Preventing peptidoglycan formation is lethal for the organism and the bacteria die. As cell wall formation occurs during bacterial replication, beta lactam drugs only exert a bactericidal effect with replicating organisms and not sessile or dormant cells [12]. In contrast, fluoroquinolone drugs target two critical enzymes necessary for bacterial replication—DNA gyrase (topoisomerase II) and topoisomerase type IV [13]. Inhibition of these enzymes is lethal to the cell and occurs in both replicating and sessile cells [14].

### 3.1. Fluoroquinolone Targets

Bacterial cells contain two quinolone targets, DNA gyrase (topoisomerase type II) and DNA topoisomerase IV [15]. Both target enzymes are trapped on DNA by quinolones as complexes that contain broken DNA. While organisms such as *Mycobacterium tuberculosis* have only DNA gyrase [16], typical Gram-negative (i.e., *E. coli*), Gram-positive (*Staphylococcus aureus*) and atypical organisms such as *Mycoplasma* species have both enzymes [17]. After the enzymes introduce double stranded breaks in the DNA, duplexes pass through the break in DNA allowing for translation after which the break is reannealed. Quinolones form complexes to either DNA gyrase or topoisomerase IV through covalently binding to the 5′ end of *GyrA* (DNA gyrase) or *parC* (topoisomerase IV) [18,19]. DNA gyrase and DNA topoisomerase IV are tetrameric A_2_B_2_ structures in which the A and B proteins are encoded by the *gyrA* and *gyrB* genes for DNA gyrase and the genes are *parC* and *parE* for topoisomerase IV. The broken DNA (cleaved complexes) [20] result from the fluoroquinolone binding to either enzyme preventing religation of the DNA substrate. DNA gyrase is responsible for negative supercoils and functionally unwind twisted DNA into a more relaxed state. Topoisomerase IV (decatenating or unlinking activity) allow segregation of catenated daughter chromosomes at cell division [21]. Quinolones result in a drug–enzyme–DNA complex trapping the enzyme. Those complexes block the movement of replication forks and transcription complexes, thereby accounting for the bactericidal effects of quinolones. It was proposed that lethal action arises from the release of the double-strand DNA breaks from the complexes [22]. Quinolones such as enrofloxacin, marbofloxacin and orbifloxacin preferentially target one of these two targets; in general, topoisomerase IV is the primary target in Gram-positive bacteria whereas DNA gyrase is the primary target in Gram-negative bacteria [23,24]. Pradofloxacin is the newest fluoroquinolone to be approved for veterinary use—first in companion animals (2011—European Union dogs and cats; 2013 USA cats) and more recently (2024) in cattle and swine. Pradofloxacin is unique in that it simultaneously targets both DNA gyrase and topoisomerase IV in both Gram-positive and negative bacteria [25].

In human medicine, the pathogen *S. pneumoniae* was a concern with some fluoroquinolones due to poor potency and the risk of resistance selection [26]. For this pathogen, some quinolones target both topoisomerase IV and DNA gyrase almost equally. Such an observation would be important because dual-acting agents should rarely enrich resistant mutants—a double mutation would be necessary for growth. Gemifloxacin and moxifloxacin were among the dual-acting agents for therapy of human infections and as mentioned, pradofloxacin for veterinary use [27,28,29]. Gemifloxacin has since been discontinued. Heaton et al. [28] grouped quinolones into the following mechanistic classes:

1. Group 1 (ciprofloxacin, levofloxacin, norfloxacin, pefloxacin and trovafloxacin (withdrawn from market in 2002))—compounds preferentially enriching mutants with alterations in the quinolone-resistance-determining region of *parC*. Such compounds are thought to act preferentially through topoisomerase IV [30,31,32,33,34,35,36,37]. Enrofloxacin, marbofloxacin and orbifloxacin most likely fit in this group.
2. Group 2 (gatifloxacin-withdrawn from market in 2006 for systemic use [27], moxifloxacin [38] and gemifloxacin [28])—compounds preferentially enriching *gyrA* mutants. Such compounds are thought to have DNA gyrase as the primary target, but they are also equally active against topoisomerase IV. Pradofloxacin would most likely fit in this group.
3. Group 3 (clinafloxacin)—an agent enriching both *gyrA* and *parC* mutants [39]. Apparently the damage caused by this compound is similar for the two enzymes. This drug is not in clinical use.

Smith et al. [40] previously questioned if safe, effective dual-targeting fluoroquinolones existed as enzymatic and genetic studies of fluoroquinolone targets do not necessarily agree. Detailed responses by Fisher and Heaton [41] and Fisher et al. [42] made the following points:

1. Quinolones act in bacteria through DNA gyrase, topoisomerase IV or both, thereby indicating dual activity [39,43].
2. Gemifloxacin and moxifloxacin exhibit dual activity based on the minimal effects that either a *parC* or *gyrA* mutation have on resistance. Strains with mutations in both target genes exhibit higher-level resistance. This is also true of pradofloxacin.
3. Enzymatic studies measure different parameters than do genetic data and need not agree.
4. Genetic data are the most reliable indicator of quinolone targets inside living bacteria.
5. Gemifloxacin and moxifloxacin act substantially through DNA gyrase and topoisomerase IV; therefore, clinically available, safe and dual-activity fluoroquinolones exist. This is also true of pradofloxacin.

Smith et al. [44] argued from clinical isolates minimum inhibitory concentration (MIC) to all compounds are low in the absence of *gyrA* and *parC* mutations; however, for *parC* mutants, the MICs to levofloxacin and moxifloxacin were elevated, as indicated by Brueggemann et al. [45] and Zhanel et al. [46] thereby indicating that the agents are not dual-acting. Fisher et al. [42] subsequently pointed out clinical isolates are heterogeneous and may carry multiple, undetected resistance mutations that affect MIC. Indeed, clinical isolates often have topoisomerase mutations whose contribution to susceptibility is undefined [47,48]. Strahilevitz and Hooper reported evidence in support of dual-targeting quinolones and reduced development of resistance [49].

### 3.2. Fluoroquinolone Resistance

Resistance to fluoroquinolones occurs by more than one mechanism. Amino acid substitutions (mutations) occurring in the quinolone resistance determining region (QRDR) change the target protein structure thereby reducing the affinity of the fluoroquinolone molecule for the enzyme target. This results in an increase in the MIC and multiple mutations further elevate MIC values rendering organisms resistant to the lethal effect of the antibiotic at clinically achievable/sustainable drug concentrations [15,50]. Mutations in the primary and secondary targets, namely *gyr*A, *gyr*B—DNA gyrase and *par*C and *par*E– topoisomerase IV leads to quinolone resistance.

Plasmid-mediated quinolone resistance (PMQR) genes are found on mobile genetic elements and *qnr* was the first to be found in a clinical strain of *Klebsiella pneumoniae* [51]. The *qnr* gene encodes a protein that binds to topoisomerase and physically prevents the fluoroquinolone molecule from binding to the target enzyme [52]. Multiple *qnr* genes have been described (*qnrA* Original), *qnrB*, *qnrS*, *qnrC*, *qnrD* [53,54]. The degree of elevated MIC values varies amongst carriage of *qnr* genes and is likely due to both plasmid copy numbers and gene expression. A variant PMQR gene from aminoglycoside acetyl transferase [aac(6′)-1b-cr] can degrade some fluoroquinolone compounds [51].

Altered drug accumulation affects the intracellular drug concentration. As quinolones interact on intracellular enzyme targets, penetrating the cells in sufficient concentrations is essential for antimicrobial activity. Reduced intracellular drug concentrations can occur by three mechanisms. First, active drug transport from the cell. For *S. aureus*, the efflux pumps NorA [55], NorB [56] and NorC [57] are responsible for four- to eight-fold increases in resistance to quinolones. These transporters have also been characterized in other Gram-positive bacteria and are reviewed elsewhere [58]. Gram-negative efflux pumps are in the Resistance–Nodulation–Division (RND) and confer quinolone resistance using pumps with three structural components (pump protein in cytoplasmic membrane, outer membrane channel protein, membrane fusion protein linking the pump and outer membrane protein) [59]. The best studied to date is the AcrAB-TolC pump complex of *E. coli*. A more detailed review has been previously published [58]. Second, reduced quinolone uptake due to a reduction in outer membrane diffusion channels and third, a combination of efflux and reduced uptake.

There is little doubt that we have a global pandemic of antimicrobial resistant microorganisms with some organism/antimicrobial issues being more prevalent than others. Despite regional variations in resistance rates for various bacteria/drug combinations, the overall impact of this trend has impacted (in human medicine) on individual patients, the economics of managing infections as well as our approach to the empirical use of antimicrobial compounds for both inpatient and outpatient management. In animals, antimicrobial resistance can impact therapy in individual animals as well as herd health and metaphylaxis protocols [60]. Indeed, various treatment guidelines (human, companion animals) from expert working groups [61,62] recommend different treatment options based on the likelihood of resistant pathogens and the need for frequent updates on these recommendations may parallel changing resistance rates. Similarly published antimicrobial guidelines for food animals are lacking. Guidelines for the treatment of companion animal infections have been published for urinary tract infections [63,64], respiratory tract infections [65,66] and dermatology [67].

Antimicrobial resistance initially was thought to be primarily an issue with nosocomial infections; however, toward the latter part of the 1980s and early 1990s both community-acquired respiratory and urinary pathogens began to exhibit antimicrobial resistance profiles [68]. In human medicine, escalation in the prevalence of antimicrobial resistance for pathogens such as *Streptococcus pneumoniae*, *Haemophilus influenzae*, *Moraxella catarrhalis*, *Escherichia coli* and *Staphylococcus aureus* led to the utilization of different, sometimes broader spectrum, and often more expensive antimicrobial agents for community-acquired infections. Similarly, in the hospital environment the escalation of resistance with enteric Gram-negative bacilli (*E. coli*, *Klebsiella* spp., *Proteus* spp. etc.), *Pseudomonas aeruginosa*, *Staphylococcus* species and *Enterococcus* species has substantially reduced the number of agents available for many infections. Increasing antimicrobial resistance is not a problem restricted to human pathogens. Indeed, increasing antimicrobial resistance is also problematic in veterinary medicine and extends to organisms such *Escherichia coli*, *Pseudomonas aeruginosa*, *Staphylococcus pseudintermedius*, *Mannheimia haemolytica* and *Pasteurella multocida*. Since the mid 1990s, relatively few new antimicrobial compounds were in clinical development in either of human or veterinary medicine and as such it became and remains essential to design strategies to prevent further antimicrobial resistance escalation. A recent comprehensive review on antimicrobials and antimicrobial resistance highlighted difficulties with new drug discovery and the need for alternative antimicrobial approaches [69].

## 4. Pharmacokinetics/Pharmacodynamics

Pharmacokinetics (PKs) define the fate of the drug in the body and considers absorption, transformation, distribution and elimination. Pharmacodynamics (PD) define the effect of the drug on the body and an infectious agent and considers the mechanism of action and drug efficacy [70]. PKs/PDs define pharmacological principals and considering an MIC, mutant prevention concentration (MPC—discussed in detail in Section 6.3) or kill measurement without PK/PD data is of little or no value. Pharmacokinetic variables for veterinary fluoroquinolones in various animals are summarized in Table 3.

Schentag [87] and others combined antimicrobial activity with fluctuating drug concentration to estimate drug exposure. Fluoroquinolones are considered to be concentration-dependent agents and the maximum serum concentration (C_max_) to MIC ratio (C_max_ /MIC) reflects the antibacterial effect as does the area under the drug concentration-time curve (AUC) that is above the MIC (AUC/MIC)—a ratio referred to as the area under the inhibitory curve (AUIC or AUIC/MIC) [87].

**Figure 3:**
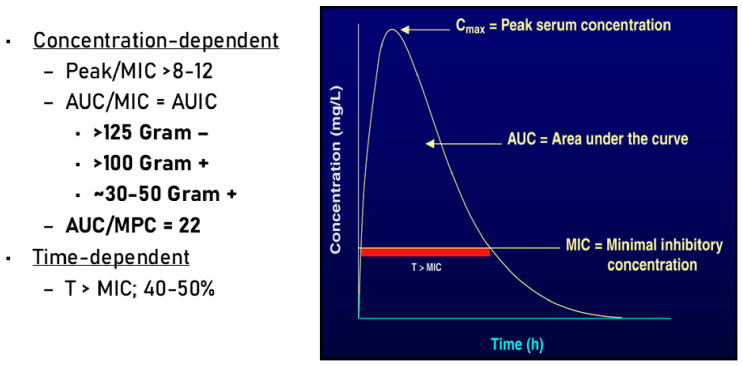
Schematic representation of a drug curve highlighting the peak drug concentration and area under the drug concentration curve in reference to the MIC.

Figure 3 is a schematic representation of a drug curve. Highlights include a peak or maximum drug concentration (serum or tissue) and the trough concentration with the drug half-life determining the dosing frequency. A C_max_/MIC ratio of >8–12 has been argued to impact clinical outcome and reducing the likelihood for resistance selection; an AUC/MIC ratio of >125 has been argued to be a necessary target value. In a report by Forrest et al. [88] from severely ill patients in an intensive care unit, a favorable clinical outcome was observed with an AUIC > 125 for both Gram-positive and Gram-negative bacteria. Further investigations with the pathogen *S. pneumoniae* suggested an AUC/MIC value between 30 and 50 might be sufficient [89], although controversy exists around this lower value [87,90,91].

At least one clinical study suggests a higher AUC/MIC ratio is beneficial for Gram-positive pathogens as well. File et al. studied human patients with chronic lung diseases and that had infectious exacerbations [92]. For patients treated with an agent for which the AUC/MIC was <100, these patients were statistically more likely to go on to develop pneumonia than were patients treated with agents where the AUC/MIC was >100. That study suggests a clinical benefit in the higher AUC/MIC. One study has suggested from in vitro investigations with ciprofloxacin and *E. coli* that an AUC/MPC ratio of ≥22 was necessary for resistance prevention.

With respect to resistance, it is important to emphasize that one limitation to PK/PD modeling relates to MIC values of susceptible bacteria and currently does not address approaches to restricting mutant growth; however, at least one study, to date, attempts to relate PD parameters with MPC measurements. Zhang et al. reviewed pharmacodynamic parameters of PK/PD integration models. They do suggest that accuracy in determining MIC can be a challenge and influence the therapeutic effect [93]. Part of this review was modeling to prevent drug resistant mutations. Balaje reported on MPC and PK/PD relationships for enrofloxacin (12 mg/kg) with *P. multocida* in buffalo calves [94]. The MIC, minimum bactericidal concentration (MBC) and MPC were 0.05, 0.06 and 1.50 µg/mL respectively. The authors indicated PK/PD data in conjunction with MIC_90_ and MPC data predicted dosages for enrofloxacin that may achieve optimum efficacy for bacteriological/clinical cure and minimized risk of resistance emergence. Jaganath and colleagues commented on MPC and incorporation with pharmacokinetic studies to more accurately determine appropriate drug concentration targets in children to restrict resistant mutant growth with treatment for tuberculosis [95].

Bergogne-Berezin (2002) reminded us the primary objective of antimicrobial agents is to treat infectious disease and Pankely and Sabath stated “Antimicrobial therapy, a keystone in modern medical practice, provides one of the only pharmacologic treatments that cure disease” [96]. Regardless, several factors affect the fate of antibacterials in the body including (1) site of infection, (2) general condition of the patient and (3) ability of drugs to reach and maintain effective concentrations in the body at infected sites—dependent on physicochemical properties of antibacterials—which govern their PK/PD parameters [97]. Regarding protein binding, an important physicochemical characteristic of antibacterials—Bergogne-Berezin suggested that it was a controversial parameter and of questionable clinical significance; however, others disagree [97]. Specifically, regarding fluoroquinolones, protein binding ranges (in general) from 20 to 40% in plasma and is bound predominantly to albumin. The author suggested protein binding with these compounds appears to be complex but has no clear role in therapeutic effectiveness or toxicity with fluoroquinolones despite early observations of such associations appearing to be important for Beta-lactam agents [98].

It is clear that in humans/animals being treated with antimicrobials, several possible outcome scenarios may occur: (1) clinical resolution and complete pathogen eradication, (2) clinical resolution with organism persistence, (3) clinical resolution with pathogen persistence and reduced susceptibility or resistance to the treatment antimicrobial, (4) clinical failure with organism proliferation, (5) clinical failure with proliferation of an antimicrobial resistant pathogen and (6) clinical failure due to infection with secondary or mixed pathogens.

For optimal antimicrobial therapy, scenario #1 is ideal but as previously discussed the measurements for determination of MIC and MPC values are different; a MIC determines the minimum amount of drug required to inhibit the growth of 10^5^ organisms/mL whereas MPC determines the antimicrobial drug concentration required to block the growth of the least susceptible cell present in bacterial inocula ≥10^9^ CFUs. Does it, therefore, seem likely that a different AUC/MPC value would apply for resistance prevention against a Gram-positive organism versus a Gram-negative organism? Unfortunately, we do not yet have an answer to this question. Zinner et al. using an in vitro pharmacodynamic model tested moxifloxacin against the human respiratory pathogen *S. pneumoniae* and suggested that an AUC_24_/MIC >100 h may protect against selection of resistant *S. pneumoniae* mutants [99]. Unfortunately, this value does not tell us what the AUC/MPC value needs to be. Metzler and colleagues determined MIC and MPC values for methicillin-susceptible strains of *S. aureus* tested against gatifloxacin, gemifloxacin, levofloxacin and moxifloxacin [100]. AUC_0–24_/MPC_90_ ratios were calculated for total and free drugs and were as follows, respectively: 51.3/41, 16.8/6.7, 48/35.5 and 190/119.7. Unfortunately, we remain unsure as to what these values actually mean given that specific studies to investigate what the AUC/MPC values for *S. aureus* and fluoroquinolones needs to be have yet to be determined. Similarly, Blondeau et al. calculated AUC_0–24_/MPC_90_ ratios for the same four fluoroquinolones against clinical isolates of *Streptococcus pneumoniae* [101]. Those values respectively were 26.7, 18.4, 12 and 47.5: free drug values respectively would be 21.4, 7.4, 8.8 and 29.9. Once again, it remains unclear as to the significance of these values given that the appropriate studies have yet to be completed to determine what the AUC/MPC values need to be.

Homma and colleagues using an in vitro pharmacodynamic model accessed the AUC/MPC for levofloxacin and moxifloxacin to prevent the emergence of resistant mutant *S. pneumoniae* [102]. When the AUC/MPC or C_max_/MPC was above 13.41 and 1.20 respectively, complete organism susceptibility was not seen. Lower AUC/MPC or C_max_/MPC values were associated with a decrease in organism susceptibility. Liang et al. reported MPC-based PK/PD indices (AUC/MPC > 25; C_max_/MPC > 2.2) for predicting mutant-restricting fluoroquinolones doses when considering levofloxacin-resistant subpopulation of *S. aureus* [103].

Olofsson et al. investigated the selection of ciprofloxacin resistance in *E. coli* in an in vitro kinetic model to determine the relationship between drug exposure and MPC [104]. In this study, two ciprofloxacin-susceptible strains and one strain containing a first-step DNA gyrase mutation were evaluated. The parameters investigated included time >MPC (T > MPC), C_max_ and AUC/MPC. The authors concluded that neither of T > MPC nor C_max_ proved to be single correlates for preventing resistance development in their experiments and against the strains tested. Against the two wild type susceptible strains, the authors found that an AUC/MPC ratio of ≥22 was a single pharmacodynamic index that predicted prevention of resistant mutant amplification. The authors also concluded that further studies are warranted to verify the usefulness of this pharmacodynamic index for the design of dosing regimens. To the best of our knowledge, similar types of experiments have yet to be published related to Gram-positive pathogens. As such, it remains unclear if a different AUC/MPC value is necessary for Gram-positives versus Gram-negatives as has been argued for AUC/MIC values.

With veterinary pathogens, Blondeau and Fitch determined MIC and MPC values for enrofloxacin, marbofloxacin and pradofloxacin against field isolates of *A. pleuropneumoniae* (n = 29) and *P. multocida* (n = 41) from swine [105]. For *A. pleuropneumoniae*, the MIC_90_ and MPC_90_ values were enrofloxacin 0.031/0.5 µg/mL, marbofloxacin 0.031/0.25 µg/mL and pradofloxacin ≤0.001/0.063 µg/mL and respectively the AUC/MIC_90_ and AUC/MPC_90_ values were as follows: 1543/995.7, 1005.5/124.7 and 1075/273. For *P. multocida* the MIC_90_ and MPC_90_ values were enrofloxacin ≤0.016/0.125 µg/mL, marbofloxacin ≤0.016/0.125 µg/mL and pradofloxacin ≤0.016/0.031 µg/mL. The AUC/MIC_90_ and AUC/MPC_90_ values were as follows: enrofloxacin 2991.3/389.2, marbofloxacin 1948.1/249.4 and pradofloxacin 1075/554.8. Gebru and colleagues investigated *E. coli* strains of canine origin against enrofloxacin and marbofloxacin in an in vitro dynamic model where a range of simulated AUC curves over 24-h intervals were measured [106]. They reported maximum losses in fluoroquinolone susceptibility occurred at AUC_24_/MIC ratios between 40 and 60. An AUC_24_/MPC ratio of 39 (enrofloxacin) and 32 (marbofloxacin) was considered to be protective against the selection of resistant mutants of *E. coli*.

One could argue that optimal antimicrobial therapy would be that which yields a favorable clinical outcome, eradicates the infecting pathogen and minimizes the likelihood for resistance selection during drug exposure. However, for some drugs, such an approach may be prevented by adverse events observed at higher, but microbiologically necessary drug concentrations. Unfortunately, use of antimicrobial drugs which fail to cure without resistance selection will invariably drive higher costs overall as prescribers will need to use more expensive and/or broader spectrum drugs to treat resistant pathogens. Treatment guidelines for a variety of infectious diseases are beginning to address a new approach to prescribing antibiotics based on a century old concept; that of hitting hard and hitting fast [107]. Shorter durations of therapy where possible using the highest, safest antibiotic drug concentration may possibly provide favorable clinical outcomes, minimal side effects and preserve drug classes for future patients. Application of PK/PD to MPC concepts to avoid selecting mutant strains within bacterial populations can help improve both short term and long-term outcomes.

The idea of “best-in-class” has been suggested as a potential way to optimizing therapy, maximize successful patient outcomes while minimizing resistant mutant selections. Once the class of agent to be used for therapy has been decided, the most potent (microbiologically, pharmacologically) agent in the class would be used based on the organism(s) most likely to be the cause of infection. One major problem with this approach is that patients are most often treated empirically; consequently, microbiological investigations may not be performed to identify the pathogen(s) and its susceptibility profile. In one study, physicians did not order any tests prior to antibiotic prescribing in 21% of urinary tract infections (urinalysis, urine culture, blood culture) and 30% of pneumonias (x-ray, sputum culture, blood culture) [108]. A fundamental problem is that two compounds can have very similar activity with susceptible cells but very different activities against resistant mutants [29,47,109]. A mutant-active agent is more likely to restrict enrichment of mutant subpopulations and ideally, best-in-class should include anti-mutant activity and not be based exclusively on MIC measurements.

## 5. Fluoroquinolones/Drug Interactions

Drug interactions with compounds containing divalent and trivalent cations (e.g., iron, aluminum, calcium, magnesium, zinc) can reduce quinolone absorption from the gastrointestinal tract and reduce bioavailability. In humans, some fluoroquinolones (enrofloxacin and ciprofloxacin) impair the liver metabolism of theophylline and caffeine. Co-administration of quinolones and non-steroidal anti-inflammatory drugs may inhibit gamma-aminobutyric receptors in the brain.

## 6. In Vitro Susceptibility Measurements

Johnson commented on the predictive value of in vitro clinical methods used to evaluate antimicrobial efficacy [110]. For hosts with normal immune defences, an organism susceptible to an antimicrobial agent, as indicated by a low MIC has a predictive value for a favorable outcome whereas antibiotic resistance as indicated by a high MIC is usually predictive of an unfavorable outcome. Additionally, higher MICs usually indicate a greater likelihood of clinical failure. Similar points were made by Johnson regarding hosts with endocarditis, meningitis or deficient immune defences. This, therefore, suggests that the in vitro measurement of antimicrobial susceptibility has utility in clinical practice [110].

Measurements of in vitro susceptibility or resistance of bacteria to antimicrobial agents is by standardized methodologies. MIC can be determined by micro-broth dilution, agar dilution or by E-test. The MIC drug concentration is the globally accepted measurement of susceptibility or resistance as the MIC value can be considered in light of drug pharmacological properties and decisions regarding longer term versus shorter-term therapy. As an example and considering the drug ciprofloxacin and the bacteria *E. coli*, a strain with an MIC of 0.008 µg/mL compared to a strain with an MIC of 0.25 µg/mL are both considered susceptible based on current breakpoints; however, these organisms are quite different. Disk diffusion (Kirby–Bauer) susceptibility testing is based on inoculating 10^5^ cfu/mL to an agar plate, upon which is placed a drug-impregnated disc or an e-test strip with a known range of drug concentrations. Following incubation under controlled conditions (temperature, atmosphere, duration) the diameter of the zone of inhibition around the disc is measured, or for e-test, the point of intersection of organism growth and antibiotic concentration can be read as the MIC. Zone sizes greater than a certain diameter are considered susceptible whereas zone sizes less than a certain diameter are considered non-susceptible. Breakpoints or drug concentration values used to determine susceptibility or resistance to a particular drug have been established by two globally recognized groups—the Clinical and Laboratory Standards Institute (CLSI) (www.clsi.org) (URL accessed on 1 June 2026) and the European Committee for Antimicrobial Susceptibility Testing (EUCAST) (www.eucast.org) (URL accessed on 1 June 2026). Breakpoints are established by considering the in vitro activity of the drug against target pathogens and the drug’s PK/PD properties in veterinary medicine—animal species.

As stated, standardized susceptibility testing has been useful and does serve as a guide for the management of most patients with infectious diseases. Exactly which patients benefit most from susceptibility testing remains debatable (i.e., outpatients with self-limiting mild to moderate infection vs. inpatients with sepsis). Determining which patients benefit from antimicrobial therapy may not be easy to resolve. It is important to recognize, however, that the correlation between an in vitro susceptibility result and clinical outcome is not 100% as other factors play a role in determining outcomes. Indeed, patients treated with a seemingly appropriate antimicrobial (i.e., the organism is susceptible) may still clinically fail to respond while those treated with an inappropriate antibiotic (i.e., the organism is resistant) may still show a favorable clinical response. In some situations, a breakpoint may not fully represent achievable drug concentration in all compartments (i.e., serum vs. urine) [111]. While the explanations for these observations are likely quite complicated, the simple explanation is that response to infection is greatly influenced by the host and the presence or absence of a competent immune response. Antimicrobial compounds remain an adjunct therapy to the body’s own natural defences [112].

### 6.1. Fluoroquinolones and Mannheimia haemolytica and Pasteurella multocida

Considerable interest has emerged regarding optimal antimicrobial therapy in both food and companion animals with acute infection requiring treatment. Indeed, most issues relevant to human infectious diseases and therapy also apply to infections in animals.

Greko et al. studied the PK/PD relationship of danofloxacin against *M. haemolytica* in a Tissue-cage model in calves [113]. The PK/PD index that was the best predictor of the antimicrobial effect was the AUC/MIC ratio.

Enrofloxacin (5 mg/kg) was compared to marbofloxacin (2 mg/kg). For clinical isolates of *E. coli* (n = 29–1786), MIC_50_ and MIC_90_ values for enrofloxacin ranged from 0.016 to 0.03 and 0.06 to 0.25 µg/mL as compared to 0.016 to 0.06 and 0.06 to 0.25 µg/mL for marbofloxacin. By comparison of MIC_90_ values for enrofloxacin against *Klebsiella* spp. (n = 55), *S. aureus* (n = 169), *S. agalactiae* (n = 29), *S. dysgalactiae* (n = 65), *S. uberis* (n = 102) and *A. pyogenes* (n = 43) were 0.03, 0.25, 2, 1, 1, 1 µg/mL respectively and for marbofloxacin, those values respectively were 0.03, 0.5, 2, 2, 2, 2 µg/mL.

Grobbel et al. reported on MIC values for various Gram-negative and positive organisms tested against six fluoroquinolones (enrofloxacin, ciprofloxacin, marbofloxacin, dorafloxacin, difloxacin and norfloxacin) [114]. In comparing MIC_50_ data for enrofloxacin and ciprofloxacin, overall susceptibility was better for ciprofloxacin against isolates of *M. haemolytica*, *P. multocida* and *E. coli*. Ciprofloxacin MICs were also lower than those for the other quinolones tested for the above noted organisms. Pirro et al. (and McKeller et al. showed that enrofloxacin and ciprofloxacin have additive activity when tested together in in vitro assays [115,116].

Previously, Blondeau et al. [117] reported that in general, the lower the MIC, the lower the MPC with *M. haemolytica*: MPC values were ≤0.5 µg/mL for organisms with MICs to enrofloxacin of ≤0.12 µg/mL. Clearly, the enhanced potency of ciprofloxacin against *M. haemolytica* strain would also yield correspondingly lower MPC values. Hansen et al. reported for clinical isolates of *E. coli*, an MPC value of ≤0.5 µg/mL [118]. More recently, Blondeau and Fitch reported on MIC and MPC values for eight antimicrobial agents (including three fluoroquinolones) against swine isolates of *A. pleuropneumoniae* (n = 29) and *P. multocida* (n = 41) [105]. All isolates needed to be susceptible (by MIC testing) to all antimicrobial agents for inclusion in the study to determine MPC values. With *A. pleuropneumoniae*, MIC_50_, MIC_90_ and MIC_100_ values for enrofloxacin were 0.031, 0.031 and 0.25 µg/mL as compared to marbofloxacin 0.031, 0.031 and 0.125 µg/mL and pradofloxacin ≤0.016, ≤0.016 and ≤0.016 µg/mL. By MPC testing the MPC_50_, MPC_90_ and MPC_100_ values were as follows, respectively: enrofloxacin 0.25, 0.5, 1 µg/mL, marbofloxacin 0.063, 0.25, 1 µg/mL and pradofloxacin 0.031, 0.063 and 0.125 µg/mL. For *P. multocida* the MIC_50_, MIC_90_ and MIC_100_ values were enrofloxacin ≤0.016, ≤0.016 and ≤0.016 µg/mL, marbofloxacin ≤0.016, ≤0.016 and ≤0.016 µg/mL and pradofloxacin ≤0.016, ≤0.016 and 0.031 µg/mL: MPC_50_, MPC_90_ and MPC_100_ values, respectively, were enrofloxacin 0.063, 0.125 and 0.125 µg/mL, marbofloxacin 0.063, 0.125 and 0.25 µg/mL and pradofloxacin ≤0.016, 0.031 and 0.063 µg/mL.

Blondeau et al. analyzed 285 strains of *M. haemolytica* collected from cattle with BRD in the USA [119]. The MIC_50_ and MIC_90_ values for enrofloxacin were 0.016 µg/mL and 0.125 µg/mL with corresponding MPC_50_ and MPC_90_ values of 0.25 µg/mL and 1 µg/mL. As MIC values become elevated, the MPC values are correspondingly elevated as well. In a different report [120], 18 clinical isolates of *P. multocida*, also collected from cattle with BRD in the USA, were analyzed by MIC and MPC testing. The MIC range values were from 0.008 to 0.5 µg/mL and the MPC_50_ and MPC_90_ values were 0.031 and 0.125 µg/mL, respectively. For *M. haemolytica*, dosages of enrofloxacin of 7.5 mg/kg and 12.5 mg/kg would yield serum drug concentrations that were above the mutant selection window (MSW) for 16–24 and 17–24 h respectively. In comparison, the time serum drug concentrations would be expected to remain above the MSW for *P. multocida* with the above noted dosages would be >24 h. This data suggested enrofloxacin demonstrates a lower propensity to select for resistance with *M. haemolytica* and *P. multocida* strains—an observation also noted for pradofloxacin.

Blondeau et al. also tested *M. haemolytica* strains collected from cattle with BRD in the USA that were collected in 2006 [121]. A total of 61–66 isolates were tested by MIC and MPC measurements. The MPC_90_ value for enrofloxacin was 0.25 µg/mL with serum drug concentrations expected to be above the MSW ≥22 h.

In a more recent study with pradofloxacin, enrofloxacin and marbofloxacin and considering C_max_/MIC_90_, C_max_/MPC_90_, AUC/MIC_90_ and AUC/MPC_90_ ratios for the three fluoroquinolones tested, the following observations were seen. With *A. pleuropneumoniae*, the ratios respectively were enrofloxacin 35.5, 2.2, 1543.9 and 95.7, marbofloxacin 48.4, 6.4, 1005.5 and 124.7 and pradofloxacin 165, 41.9, 1075 and 273. For *P. multocida* those values, respectively, were 68.75, 8.8, 2991.3 and 382.9, marbofloxacin 100, 12.8, 1948.1 and 249.4 and pradofloxacin 165, 85.2, 1075 and 554.8 [105].

In a study published by Blondeau and Fitch, clinical isolates of *Pasteurella multocida* (n = 90) were tested by MPC against enrofloxacin and other agents [122]. MPC_90_ values for enrofloxacin were 0.125 µg/mL. Enrofloxacin had low MPC_90_ value against strains of *Actinobacillus pleuropneumoniae* at 0.5 µg/mL.

Pradofloxacin has in vitro activity against anaerobic organisms and clinical outcome data support its use for adjunctive therapy in indications where anaerobic organisms are potentially problematic (i.e., periodontal infections) [123].

Therefore, what have we learned from MPC measurements conducted and published from our laboratory is as follows:

1. As the bacterial density increases, higher drug concentrations are required to inhibit 100% growth of the bacterial cells.
2. Two or more compounds within the same drug class with similar MIC values against a particular pathogen may have very different MPC values.
3. Higher MPC values exceeding maximum serum or tissue drug concentrations cannot be achieved with conventional dosing and as such use of the drug likely facilitates the selection and amplification of bacterial sub-populations that are resistant to the treatment antibiotic.
4. Examples of selection of drug-resistant bacteria to the treatment antibiotic are published [124,125].

A drug within a particular class that more readily selects for drug resistant subpopulations may be a “class killer” in that once resistance is selected in a particular pathogen, that pathogen is now resistant to all compounds within the drug class [126].

It is often unclear whether microbiological data should play in clinical decision making. The vast majority of antimicrobials are prescribed empirically—that is, without specific pathogens or pathogen susceptibility being known. One study from South Africa reported ≥70% of general practitioners prescribed antibiotics empirically—primarily for symptom relief and prevention of complications in humans. In veterinary medicine, 71% of veterinarians prescribed antimicrobials empirically and in >30% of cases and 21.5% respectively never or rarely requested culture and susceptibility data due to financial constraints, delay in treatment and time constraints [127]. Of interest in this study, 85% of respondents did not have formal antimicrobial use guidelines in their clinic; 82% cited costs as the main obstacle for antimicrobial susceptibility testing and 80% avoided antimicrobial agents in clean surgeries. When microbiological data become available, they may or may not initiate a change in patient management; for patients clinically responding, there may be a reluctance to change antimicrobial therapy—particularly if there are costs associated with a change in therapy. That may then have a negative impact by enriching resistant mutant subpopulations. With patients having mild-to-moderate disease, an additional problem arises when specimen collection is difficult or inconvenient: specimens are often not collected. In veterinary medicine, specimen collection for culture and susceptibility testing may be discouraged due to costs. These patients are treated empirically and respond clinically; however, therapy may have been suboptimal, thereby contributing to enrichment of mutant subpopulations—a favorable clinical response does not necessarily mean the pathogen has been completely eradicated and that resistant mutants were not selected. Antimicrobial therapy choices are often driven by costs; price has nothing to do with science.

### 6.2. Bactericidal Versus Bacteriostatic

The definition of bactericidal and bacteriostatic is problematic in that it is historically based on testing of a bacterial inoculum of 10^5^ cfu/mL. This organism density may not necessarily be representative of organism density that occurs during human and animal infections. For example, in humans with central nervous system infections such as meningitis [128], respiratory tract infections [129,130], urinary tract infections [29,47,109,110,111,128,129,131,132,133,134,135], skin and skin structure [136] and possibly others, bacterial densities during infection may range from 10^2^-10^9^ CFU/mL or gram of tissue or total CFUs. In animals, high density bacterial numbers were reported from the respiratory tract [130,137], urinary tract [138] and likely other anatomical locations with acute bacterial infections. Frisch et al. published that in human patients with pneumonia caused by *S. pneumoniae,* the total bacterial burden may exceed 10^12^ bacterial cells [129]. As such, a definition of bactericidal versus bacteriostatic activity based on low bacterial densities is limited and may be misleading regarding the amount of drug necessary to block total bacterial population growth. Blondeau et al. reported that for macrolide compounds (azithromycin, clarithromycin, erythromycin) exposed to higher densities of bacteria in kill assays, they tended to display bactericidal properties, yet at lower densities they exhibit bacteriostatic properties based on the classic definition [139].

Blondeau and Fitch compared the in vitro killing of swine isolates of *P. multocida* against eight antimicrobial agents of which three were fluoroquinolones (summarized in greater detail later in the manuscript) [140]. Bacterial densities from 10^6^ to 10^9^ cfu/mL were exposed to four clinically relevant drug concentrations (MIC, MPC, C_max_ and maximum tissue drug concentration) and the log_10_ reduction and percent kill of bacterial cells measured at timed intervals over 24 h. At the maximum serum drug concentration, enrofloxacin, marbofloxacin and pradofloxacin at a 10^6^ cfu/mL density resulted in a <3 log_10_ reduction in viable cells (81.7–98.9% kill). Similarly at the 10^7^ cfu/mL density a 1.5–2.9 log_10_ reduction in viable cells resulted in a 93.5–99.8% kill. This data further questions the definition of bactericidal based on in vitro measurements and what log_10_ reduction is required. As such, the definition of bactericidal versus bacteriostatic may be dependent on the Bug–Drug combination and the density of organisms and drug concentrations tested. In human medicine, and where comparative data exists, demonstrating differences in clinical outcome based on cidal versus static drugs has not been seen.

### 6.3. Mutant Prevention Concentration (MPC)/Mutant Selection Windows (MSWs)

The MPC defines an in vitro measurement—conceptually similar to MIC testing—but with a much higher density of organisms. The concept for MPC testing arose from the observations that spontaneous mutants arise in bacterial populations of ≥10^7^ CFU/mL and that patients get infected with bacterial densities of 10^7^ CFU/mL or greater [101,128,130,141,142,143]. Indeed, for most Bug–Drug combinations, spontaneous mutants arise over densities of 10^7^–10^9^ CFUs and as such MPC testing is done on bacterial densities of ≥10^9^ CFUs, whereas MIC testing is based on 10^5^ CFU/mL—a density too low to detect the presence of spontaneous mutant cells that arise at clinically relevant bacterial densities. From initial measurements with fluoroquinolones and *S. aureus* and *Mycobacteria smegmatis* strains, it was found that as the density of bacterial cells exposed to drug in vitro increased, two distinct drug concentrations inhibiting bacterial growth were recognized. The first approximated by the MIC drug concentration, above which at higher drug concentrations, colonies were isolated on drug containing agar plates. When analyzed for amino acid substitutions in the QRDR, mutations conferring reduced susceptibility or resistance to the fluoroquinolone were found. Drug concentrations blocking growth of mutant cells was termed the mutant prevention concentration. It is important to point out that MPC is not mutation prevention as antimicrobials neither induce nor prevent mutations from occurring. Rather, the MPC measurement is the drug concentration preventing mutant growth. Typically, MIC drug concentrations are lower than MPC drug concentrations indicating prevention of growth of mutant subpopulations from high density bacterial, and inocula in vitro requires higher drug concentrations. MPC testing is technically more demanding than MIC testing and as such is not yet ready for routine diagnostic testing. To measure the MPC, ≥10^9^ CFU are applied to the surface of agar plates containing antimicrobial agent tested in doubling dilutions (as with MIC testing) and incubated and read at 24 and 48 h with the MPC defining the antimicrobial drug concentration blocking 100% growth. With fluoroquinolones where resistance arises from spontaneous mutants in genes (*parC*, *gyrA*) encoding for the quinolone target proteins topoisomerase IV and DNA gyrase, MPC was defined as the drug concentration necessary to block growth of first step resistance mutants. As MPC measurements expanded to other drug classes, the definition was to block the growth of the least susceptible cell present in high-density bacterial populations regardless of mechanism (mutant, tolerance, etc.). A summary of MPC data for various organisms/antimicrobial agents was summarized previously [144].

Many bacterial strains readily grow to high densities; however, for some bacterial species achieving a high enough inoculum to deliver 10^10^ cells to each of a series of drug-containing plates is difficult and a centrifugation step to concentrate bacterial cells is used [101,134]. Huang and co-investigators determined MIC and MPC values for danofloxacin and some non-fluoroquinolone antibacterial agents against *Mycoplasma hyopneumoniae* (ATCC 25934) [145]. Danofloxacin MIC was 0.125 µg/mL, MIC_99_ 0.101 µg/mL and the MPC was 1.0 µg/mL with a selection index (MPC/MIC_99_) of 9.90.

**Figure 4:**
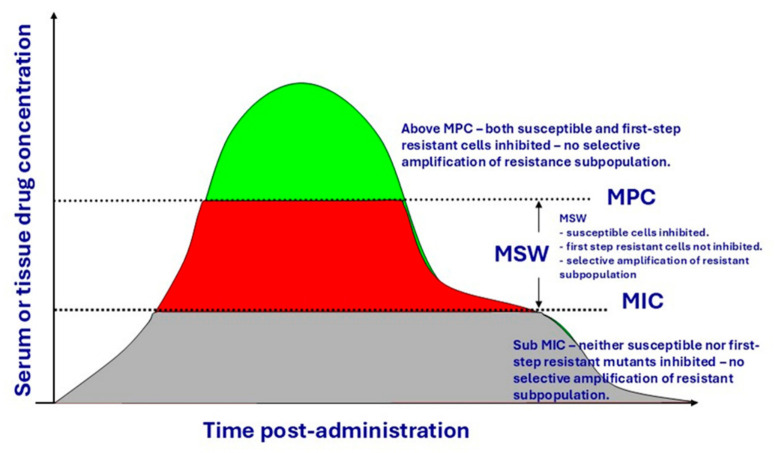
Schematic representation of the mutant selection window showing the lower (MIC) and upper (MPC) boundaries. Reproduced with permission Journal of Chemotherapy, 2004, 16 (Suppl.3): pg 1–19 [101].

The MSW defines a drug concentration range in which resistant mutants are selectively enriched (Figure 4) in the presence of drug and is bordered by the MIC (lower limit) and the MPC (upper limit) [101,134,146]. The MSW has been described as the “danger zone” for drug-accelerated enrichment of resistant subpopulations [147,148]. Antimicrobial drug concentrations falling below the MIC inhibit neither susceptible nor first-step resistant mutants. Drug concentrations above the MPC inhibit all cells and consequently prevent selective amplification of mutant cells. Between MIC and MPC drug concentrations mutant enrichment occurs as a result of susceptible cells being inhibited by drug concentrations in excess of the MIC. Arguably therapeutic drug concentrations leading to clinical cure may selectively amplify the mutant fraction from high density bacterial populations.

The MSW hypothesis has been investigated with in vitro experiments using fluctuating fluoroquinolone concentrations. Firsov et al. [149] simulated dosing of fluoroquinolones with *S. aureus* in an in vitro pharmacological model and found organisms with elevated MIC values were obtained only when drug concentrations remained within the MSW, not when above the MPC or below the MIC. Similar results were found with *S. pneumoniae* and fluoroquinolones [99]. Zhang and colleagues reported on the MSW for danofloxacin against *A. pleuropneumoniae* using an in vitro dynamic model [150]. The MIC and MPC values for the strains used were 0.0625 and 0.4 µg/mL. Using simulated time concentrations curves of danofloxacin, drug concentrations of 0.025 µg/mL and 0.05 µg/mL were located outside the MSW, 0.1 µg/mL and 0.2 µg/mL were in the lower part of the MSW, 0.4 µg/mL was located in the middle and 0.8 µg/mL was in the middle and upper parts of the MSW. For in vitro kill curves, drug concentrations of 0.025 and 0.05 µg/mL could produce a bacteriostatic effect, 0.1 µg/mL a bactericidal effect and 0.2 µg/mL an eradication effect.

Hansen et al. suggested the time drug concentrations remain in excess of the MPC may be important for restricting mutant growth [151]. In studies published with *S. pneumoniae*, a >99% reduction in viable cells occurred between 6 and 12 h of exposure to various fluoroquinolones when organisms were exposed to the MPC drug concentration in time kill experiments. Such data might suggest that time above the MSW of at least 6 h may be important for ensuring substantial reductions of high density bacterial inocula as the kill experiments highlighted above were performed using bacterial inocula ranging from 10^6^ to 10^9^ cfu/mL—inocula consistent with the MPC approach and organism densities during infection.

For bacteria other than *S. pneumoniae*, *H. somnus*, *M. haemolytica* [119] and *P. multocida* [122] growth to densities of ≥10^10^ CFU/mL occurs more readily and do not require centrifugation. Such bacteria include *P. aeruginosa* [152], *S. aureus* [153], *S. pseudintermedius* [154], *E. coli*, *Enterobacter* spp., *Klebsiella* spp. and *Citrobacter* spp. [118].

**Figure 5:**
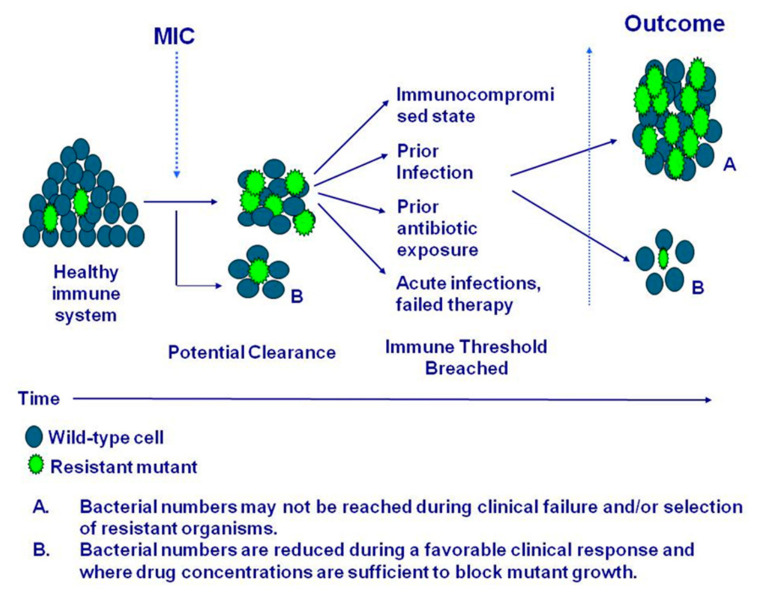
Schematic representation of resistance selection based on MIC drug concentrations. The MIC drug concentration prevents growth of organisms inhibited/killed at the concentration (wild-type) but not requiring higher drug concentrations for inhibition/kill (mutants). Figure drawn by author. Modified from Journal of Chemotherapy, 2004, 16 (Suppl.3); pg 1–19 [101].

**Figure 6:**
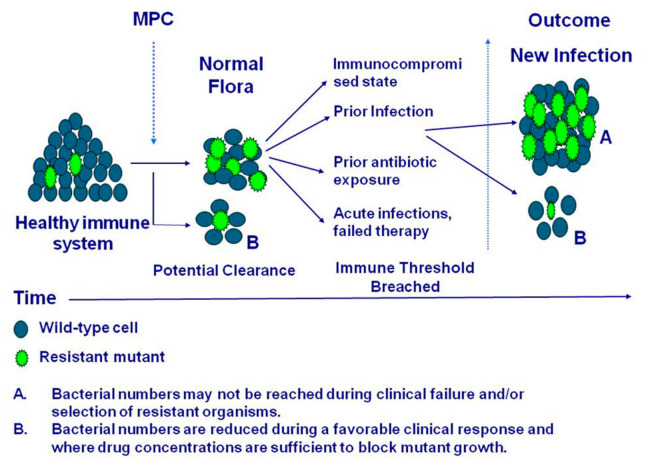
Schematic representation of resistance based on MPC drug concentrations. Figure drawn by author. The MPC drug concentration inhibits/kills wild-type and mutant cells.

Figure 5 is a schematic representation of enrichment of resistant mutants. Resistant cells present in bacterial populations containing 10^7^ or more CFU may be selectively amplified in the presence of the drug. When drug concentrations are inadequate to block mutant cell growth, proliferation in the presence of the antimicrobial agent occurs. Bactericidal drugs will dramatically reduce the susceptible percentage of a bacterial population, but resistant mutants are unlikely to be killed when drug concentrations remain within the MSW. Dosing strategies placing drug concentrations within the MSW rely on host immune systems rather than drug to remove mutant cells. Recovery from infection requires functional immunity. One concern is that repeated drug exposure inside the MSW will gradually enrich resistant mutants, especially in vulnerable patients, i.e., immunocompromised and/or those exposed to repeat sub-optimal antimicrobial therapy with the same drug or class of drugs over a relatively short period of time (i.e., 3–6 months) (Figure 6). Vanderkooi et al. (from human patients) showed an increased likelihood of having a drug-resistant organism in patients treated with macrolides, when the patient required additional therapy when seen within 3 months of their last acute complaint [155]. This unique study captured patients seen in the emergency department and who received antimicrobial therapy for invasive pneumococcal infection (n = 3339 patients). Risk factors for infection with penicillin-susceptible versus penicillin-resistant pneumococci included year of infection (*p* < 0.001), absence of chronic organ disease (*p* = 0.03), previous use of penicillin (*p* = 0.006), trimethoprim/sulfamethoxazole (TMP/SMX) (*p* < 0.001) and azithromycin (*p* = 0.05). Infection with TMP/SMX resistant *S. pneumoniae* was associated with the absence of chronic organ system disease (*p* = 0.001), previous use of penicillin (*p* = 0.03) or TMP/SMX (*p* < 0.001) or azithromycin (*p* = 0.001). Infection with macrolide-resistant pneumococci was associated with use of penicillin (*p* = 0.03), TMP/SMX (*p* = 0.04), clarithromycin (*p* < 0.001) or azithromycin (*p* < 0.001). Fluoroquinolone resistance was associated with the use of single targeting fluoroquinolone (*p* < 0.001). The authors concluded that knowledge of antimicrobial use during the previous 3 months was important for determining appropriate therapy. Arguably the same concern exists in veterinary medicine. In such situations, dissemination of enriched mutant strains will erode the utility of the agent or class of agent affected.

In immune-competent patients (depicted in Figure 5 by letter B) both drug-susceptible and drug-resistant cells are cleared. In immunocompromised patients or those with recent prior infection or with prior antimicrobial exposure or those patients that appear to be failing therapy for acute infection, it is argued that continued organism proliferation to where the immune threshold is breached may result in bacterial populations (depicted by letter A in Figure 5) with a predominance of resistant organisms; alternatively, clearance and eradication may occur as part of the overall patient response (depicted by B in Figure 5). In Figure 6, MPC based dosing drug concentrations will reduce overall bacterial numbers and prevent the selective amplification of the resistance subpopulations present as part of the total bacterial burden. Dosing strategies may not reduce the likelihood that “at risk” patients become infected with a new pathogen.

With fluoroquinolones, some mutants considered susceptible by established breakpoints may more readily acquire additional mutations than wild-type cells [48]. Such mutants would be expected to accelerate the development of resistance. As enrichment of mutants would likely go unnoticed until they are the dominant members of the population and detected by elevated MIC values. At that point they may be difficult to control. Arguably more consideration should be given to prevent enrichment of first mutants. “Best in class” (as mentioned) may be a compound or compounds that reduce the likelihood to select for resistance while remaining clinically efficacious.

Some debate has suggested that the MPC method of testing only applies to fluoroquinolone compounds [156]. Subsequently, numerous studies have elucidated MPCs against a wide variety of antimicrobial compounds and bacterial pathogens. In a previous report, an alternate terminology—resistance prevention concentration—was coined as an all-encompassing terminology to define the antimicrobial drug concentration that blocked the growth of the least susceptible organism present in high-density bacterial inoculum and was independent of the mechanism of resistance of those mutant cells [101]. In fact, MPC and RPC testing is synonymous and it remains important to remember that MPC defines the mutant prevention concentration and not the “mutation” prevention concentration.

## 7. In Vitro Kill Studies

Killing bacteria (bacteriological cure) is relevant to clinical cure. One study investigating bacteriological failure in children with acute otitis media focused on those that were culture positive before treatment. By day 4–5 of antibiotic therapy, 46% of children remained culture positive and 66% showed bacterial eradication. Of the children culture positive at day 4–5, 37% showed clinical failure by day 10 compared to 13% that showed bacterial eradication by days 4–5. Some 91% of clinical failures at day 10 were culture positive on days 4–5 of therapy. In some instances, clinical improvement may be seen; however, in patients where bacteria were not cleared, relapse may occur. Other reports comparing a bacteriostatic agent with a bactericidal agent found that eradication of the target pathogen was achieved in 33% of patients receiving a macrolide (static) as compared to 87% of those receiving a beta-lactam/beta-lactamase inhibitor (cidal) antimicrobial agent [157,158].

A dated study from the 1970s examined urinary tract infection in a human general practice population [159]. An interesting aspect of the study was a question asked specifically to patients regarding the treatment and if it made them feel better. The correlation between a patient responding cured and bacteriological cure was poor and of 134 patients responding “cure”, 74 (55%) had bacteriological cure and 60 (44%) had bacteriological failure. The explanation is likely temporary symptom relief. Patients who responded “failure” had a 91% (21/23) bacteriological failure and the answer to the question was likely influenced by persisting symptoms.

In human patients, one-day short course therapy for urinary tract infection (UTI) is associated with a higher likelihood of relapse than are longer durations of therapy—most likely due to failure to eradicate the organism [160]. Spontaneous clinical cure was found to occur in 25 (42%) of untreated women or women treated with a drug without activity against the infecting pathogen for UTI. Untreated UTI is associated with prolonged symptoms and the risk for disease progression. In one Scandinavian study, use of varying dosages of a beta-lactam antimicrobial for treatment of UTI was statistically significantly better than placebo [161]. Of 96–99% of patients free for symptoms at follow up, they were also free of bacteria; for patients with symptoms, 10–30% had persisting bacteria. At study follow up, 93–98% of antimicrobial treated patients were also bacteriologically cured whereas 25–48% of patients with symptoms also had persisting bacteria.

In vitro kill studies have been used to compare killing or inhibition of bacteria by antimicrobial agents. In such studies, defined densities of bacteria (i.e., 10^5^ cfu/mL and/or 10^6^–10^9^ cfu/mL) are exposed to various drug concentrations and the log_10_ (and % kill) reduction in viable cells recorded at varying times following drug exposure. The rate and extent of killing is measured at time intervals by comparing the reduction of viable cells from time 0. Dagan and colleagues convincingly argued that bacterial eradication is an important aim of antimicrobial therapy and necessary for clinical cure [162]. Bactericidal drugs are categorized as those resulting in a ≥3 log_10_ reduction in viable cells as compared to bacteriostatic agents where a ≤2 log_10_ reduction is seen. Reductions between two and three log_10_ was considered a “grey” zone [96,163]. One limitation with the above definition is that bacterial densities likely fluctuate during infection and during the early stages of acute infection bacterial densities may be substantially higher than 10^5^ cfu/mL.

From experiments in our laboratory, we have challenged and published [164] the approach to kill measurements and have argued kill studies need to be conducted over a range of bacterial densities (i.e., 10^5^–10^9^ CFU/mL) and tested against clinically relevant drug concentrations including the MIC, MPC, maximum serum (MS) and maximum Tissue (MT) drug concentrations (DCs). The higher bacterial densities take into account spontaneously occurring mutants that arise in bacterial densities of ≥10^7^ CFUs and the inhibition/killing of bacteria at higher densities. For example, killing of bacteria using drug concentrations that are multiples of the MIC may or may not be clinically meaningful depending on the relevance of that drug concentration to clinically achievable or sustainable drug concentrations. Testing of drug concentrations that are not clinically achievable nor sustainable does not provide clinically useful information.

As previously commented, high-density bacterial populations have been reported in both human and animal infections. How does higher bacterial densities affect bactericidal and bacteriostatic definition? Blondeau and colleagues measured in vitro killing of *S. pneumoniae* by macrolides (azithromycin, clarithromycin, erythromycin), a ketolide (telithromycin) and a fluoroquinolone (gemifloxacin) by the measured MIC and MPC drug concentrations against bacterial densities from 10^6^ to 10^9^ cfu/mL. For these experiments, the MIC and MPC drug concentration values were measured for each drug against all strain tested and those measured values were the drug concentration used in the kill assays. The kill assays were run over 24 h with measurements of kill determined at 30 min, 1, 2, 3, 4, 6, 12 and 24 h after drug exposure. For all drugs, killing at the MIC drug concentration was slower and less complete over the densities tested. At the MPC drug concentration, killing was seen with all drugs (less overall with telithromycin) and increased with the time of drug exposure. Following 2 h of drug exposure 71–98% of cells were killed by gemifloxacin versus bacterial growth of 97% for the other agents. Interestingly, at the MPC drug concentration up to 100% of bacterial cells were killed by the macrolides following 24 h of drug exposure. As previously mentioned, macrolides are typically thought of as being bacteriostatic yet against higher bacterial densities, they demonstrated bactericidal properties [96].

The killing of *Mannheimia haemolytica* strains by enrofloxacin, florfenicol, tilmicosin and tulathromycin was investigated with 1 million (10^6^) to 1 billion (10^9^) bacteria or colony forming units per milliliter (CFU/mL) exposed to the MIC, MPC, MSDC and MTDC and the reduction in viable cells recorded at time intervals. Statistical comparisons were made for the killing of the bacteria by each drug. At an organism density of 10^8^ CFU/mL exposed to the MPC drug concentration, more bacteria were killed by enrofloxacin (96%) than for florfenicol (2%, *p* < 0.0001), tilmicosin (21%, *p* < 0.0001) and tulathromycin (3.2%, *p* < 0.0001) following 30 min of drug exposure. Similar statistical differences were seen in favor of enrofloxacin at 1, 2, 4 and 6 h of drug exposure. At the 10^7^ CFU/mL density, statistically significant differences in bacterial killing were seen for enrofloxacin over florfenicol (*p* < 0.0001), tilmicosin (*p* < 0.0001) and tulathromycin (*p* = 0.0007) following 12 h of drug exposure. At the maximum serum drug concentration and bacterial densities of 10^6^–10^7^ and 10^9^ CFU/mL, statistically more bacterial cells were killed by enrofloxacin than by tilmicosin and tulathromycin at many time points and more cells were killed by florfenicol (10^6^–10^7^ CFU/mL) than by tilmicosin and tulathromycin. For example, at the 10^7^ CFU/mL, enrofloxacin killed more cells at 2, 4, 6 and 12 h of drug exposure (>99% at all times) than did florfenicol (83–99%), tilmicosin (25–76%) and tulathromycin (54–78%) with *p* values from 0.5 to 0.0001 for all comparisons. At the maximum tissue drug concentrations and a bacterial density of 10^9^ CFU/mL, enrofloxacin (90–100%) killed statistically more cells than did tilmicosin (27–75%, *p* values from 0.02 to <0.0001) and tulathromycin (22–86%, *p* values from 0.002 to <0.0001) following 1, 2, 4, 6, 12 and 24 h of drug exposure. For all drugs, longer drug exposure generally resulted in higher rates of bacterial kill. This study concluded differences existed between compounds in the killing of *M. haemolytica* strains. As well, antimicrobial drug concentrations exceeding the MPC drug concentration resulted in more rapid killing of the *M. haemolytica* strains and such observations may have important implications for reducing the selection of drug-resistant subpopulations during therapy and where high-density bacterial burdens are present during infection.

From kill experiments conducted and published from our laboratory we have learned:

(1) Killing bacteria using the MIC drug concentrations is slow and incomplete for all drugs and drug classes when higher densities of organisms are tested.
(2) Killing of bacteria using the MPC drug concentration is generally faster and with more extensive killing; however, differences emerge between killing bacteria by different drugs within the same class or between drugs in different classes. In recent publications/abstract presentations, we have applied statistical analysis to killing of bacteria by one drug versus drugs from other classes and have shown highly statistically significant differences in the speed and completeness of kill at various drug concentrations (i.e., MPC, MSDC, MTDC).
(3) Killing using the MSDC and MTDC may be faster and more complete providing these values exceed the MPC drug concentration.
(4) The standard definition of bactericidal and bacteriostatic does not apply when higher densities of bacteria are used in kill assays.

Four different canine urinary pathogens (three strains of each), namely *E. coli*, *P. mirabilis*, *S. pseudintermedius* and *E. faecalis* were exposed to the MIC, MPC and peak urinary drug concentrations of five antimicrobial agents in a 3 h kill assay. A 10^5^ cfu/mL bacterial density was used. At the maximum urine drug concentration (MUDC), significantly more *E. coli* cells were killed by marbofloxacin and pradofloxacin than by ampicillin, cephalexin and TMP/SMX (*p* < 0.0001 for all comparisons) following 5 min of drug exposure [165]. For *E. faecalis* strains, significant differences between drugs for in vitro killing was not seen at the MIC or MPC drug concentrations. At the MUDC and following 60, 120 and 180 min of drug exposure, pradofloxacin (*p* = 0.0016–0.0076) and marbofloxacin (*p* = 0.0031–0.0001) killed more cells than did TMP/SMX. For *P. mirabilis* at the MUDC pradofloxacin killed more cells than did TMP/SMX at 10 min and at various time points thereafter (*p* = 0.106-<0.0001) and cephalexin following 5, 10 and 15 min of drug exposure (*p* = 0.0483–0.0106). Similarly, marbofloxacin killed more cells than did TMP/SMX following 15 min of drug exposure and thereafter (*p* = 0.0434–<0.0001). Following 5, 10 and 15 min of drug exposure, pradofloxacin killed more cells than ampicillin (*p* = 0.004–<0.0001). Finally for *S. pseudintermedius* strains exposed to the MUDC of each drug, pradofloxacin killed more cells than did cephalexin following 20 and 30 min of drug exposure (*p* < 0.0001, *p* = 0.0474), more cells than did TMP/SMX following 60 and 120 min of drug exposure (*p* = 0.0489, *p* = 0.0178) and more cells than ampicillin following 120 min of drug exposure (*p* = 0.0348).

Silley et al. reported on the bactericidal properties of pradofloxacin against veterinary pathogens [10]. From measurements with *S. pseudintermedius*, *S. aureus*, *E. coli*, *P. multocida*, *S. canis*, *Proteus* spp., *Fusobacterium* spp., *Porphyromonas gingivatis* and *Prevotella* spp, and using a subset of these species, kill measurements were done. By minimum bactericidal concentration (MBC) testing, 27 of 30 strains had MBC values within two doubling dilutions of the MIC value. Pradofloxacin demonstrated concentration killing such that as the drug concentration increased, a faster rate of killing was seen. Bactericidal effects were seen at drug concentration ≤0.25 µg/mL in all cases examined. Bactericidal activity was also demonstrated against the anaerobic bacterial strains tested. The authors concluded rapid rates of kill will play a significant role in clinical efficacy.

Silley and colleagues reported on the comparative activity of pradofloxacin against anaerobic bacteria from dogs and cats [166]. A total of 141 anaerobic organisms were tested against difloxacin, enrofloxacin, ibafloxacin, marbofloxacin and pradofloxacin. In considering MIC_50_, MIC_90_ and mode MIC values, pradofloxacin exhibited higher in vitro activity—based on lower MIC values—than did the other drugs tested.

Azzariti et al. reported on pradofloxacin and marbofloxacin in time kill studies against canine skin pathogens (*S. aureus*, *E. coli*, *S. pseudintermedius*) [167]. They used a semi-mechanistic mathematical model to estimate the best PK/PD indices for prediction of clinical efficacy. Pradofloxacin showed a higher potency than did marbofloxacin, however, the overall killing rates (computed) were similar in most instances.

Blondeau and Fitch compared the bactericidal activity of pradofloxacin and several other antimicrobial agents for the killing of swine bacterial pathogens [168]. In this study, killing was measured in a 3 h kill assay against 10^5^ cfu/mL of strains (n = 3 for each organism) of *A. pleuropneumoniae*, *P. multocida* and *S. suis*. Considering the MSDC and MTDC, pradofloxacin was bactericidal at MSDC for *A. pleuropneumoniae* following 15–20 min of drug exposure (94.4–99.9% kill), and for *P. multocida* following 5–30 min of drug exposure 68.7–96.9%. Following 5 min of exposure to the MTDC, pradofloxacin was bactericidal for both *A. pleuropneumoniae* and *P. multocida* (91.7%). Against *S. suis* strains, pradofloxacin killed 92.4–99.4% and 71.6–97 L% of cells following 60–180 min of drug exposure (MSDC and MTDC respectively). In comparing killing between drug tested against *A. pleuropneumoniae* and following 20–60 min of drug exposure, pradofloxacin killed more cells than did enrofloxacin (*p* values 0.0015–<0.0001) or marbofloxacin (*p* values 0.0111–0.0010) at the MSDC. At the MTDC and following 30–180 min of drug exposure, pradofloxacin killed more cells than did enrofloxacin (*p* values 0.0415–<0.0001) and following 180 min of drug exposure, marbofloxacin killed more cells than did enrofloxacin (*p* = 0.0129).

The in vitro killing of swine isolates of *P. multocida* by eight antimicrobial agents (ceftiofur, enrofloxacin, florfenicol, marbofloxacin, pradofloxacin, tildipirosin, tilmicosin, tulathromycin) was previously published [140]. In this study, bacterial densities ranging from 10^6^ to 10^9^ cfu/mL were exposed to four different drug concentrations over 24 h: MIC, MPC, MSDC and MTDC. Against the 10^6^ cfu/mL bacterial inoculums and using the MIC drug concentration, killing overall was poor for most agents except marbofloxacin and pradofloxacin. By 4 h after drug exposure, marbofloxacin killed 90.5% of cells, which increased to 99.6% of cells following 24 h of drug exposure. By comparison, pradofloxacin killed 97.7% following 4 h and 99.0% of cells following 24 h of drug exposure. Similar results were seen at the MPC drug concentration and considering 4 and 24 h after drug exposure, the following kill percentages were seen, respectively: ceftiofur 75.8%/99.6%, enrofloxacin 89.5%/70.4%, florfenicol 41.9%/growth, marbofloxacin 90.9%/99.8%, pradofloxacin 96.9%/99.9%, tildipirosin 32.7%/99.9%, tilmicosin 75.7%/99.8% and tulathromycin growth/growth. At the MSDC and MTDC, killing tended to be more consistent across most drugs ranging from 98.7 to 99.4% for the three fluoroquinolones at 4 h and 100% for enrofloxacin and pradofloxacin following 24 h of drug exposure; for ceftiofur those values were 97.6%/98.1% and 99.9–100% respectively. For the other agents, killing was variable or growth occurred.

As the bacterial density exposed to antibiotics increased (10^7^–10^9^ cfu/mL), there was an impact on killing depending on the drug concentration used. In considering statistically significant observations, they only occurred following 24 h of drug exposure at the 10^7^–10^8^ cfu/mL density such that pradofloxacin killed more cells than ceftiofur (*p* = 0.0189-*p* < 0.0001), enrofloxacin (1.7 cfu/mL) (*p* < 0.0001), marbofloxacin (*p* = 0.0005–*p* < 0.0001), tildipirosin (*p* = 0.0015–*p* = 0.0003), tilmicosin (*p* = 0.0354–*p* < 0.0001) and tulathromycin (*p* = 0.0066–*p* < 0.0001). Statistically significant differences in kill were not seen between the drugs at the 10^9^ cfu/mL density; however, 99.9% of cells were killed by pradofloxacin as compared to 96.2% by enrofloxacin and 89.9% by ceftiofur.

In yet another recently published kill study, bovine isolates (*M. haemolytica* and *P. multocida*) with varying bacterial densities ranging from 10^6^ to 10^9^ cfu/mL were exposed to antimicrobial agents in a 24 h kill assay [169]. Four fluoroquinolones (danofloxacin, enrofloxacin, marbofloxacin, pradofloxacin) were compared to ceftiofur, florfenicol, tildipirosin, tilmicosin and tulathromycin based on four different drug concentrations–MIC, MPC, maximum serum and maximum tissue. Considering the four fluoroquinolone agents, killing using the MIC drug concentration was variable. As an example, for *M. haemolytica* at a 10^6^ CFU/mL inoculum, pradofloxacin killed 92% of bacterial cells following 2 h of drug exposure and 99.99% following 12 h of drug exposure. For danofloxacin and enrofloxacin, 48.2% and 65.9% respectively of cells were killed following 24 h of drug exposure. In the presence of marbofloxacin, growth occurred following 1–24 h of drug exposure. The differences in killing between pradofloxacin and the other quinolones tested was statistically different in favor of pradofloxacin at various time points investigated. For bacterial densities between 10^7^ and 10^9^ CFU/mL, killing using the MIC drug concentration declined for all drugs; however, pradofloxacin killed 91.2–96.1% of cells (10^7^ CFU/mL), 94.1–95.4% of cells (10^8^ CFU/mL), 46–95% (10^9^ CFU/mL) following 12–24 h of drug exposure. Killing by the other fluoroquinolones ranged from growth in the presence of the drug to up to 93.73% kill. Statistically significant differences were seen at some time points evaluated. At the MPC, MSDC and MTDC the quinolones were more consistent with in vitro killing. Specifically at the MPC drug concentration and considering 1 h and 12 h of drug exposure, respectively, killing was as follows for each drug: enrofloxacin, 10^6^ CFU/mL 86.8% and 99.99%, 10^7^ CFU/mL 79.8% and 90.7%, 10^8^ CFU/mL 49.8 and growth and 10^9^ CFU/mL growth; danofloxacin 10^6^ CFU/mL 35.9% and 99.5%, 10^7^ CFU/mL 9.8 and 95.6%, 10^8^ CFU/mL 25.3% and 31.7%, 10^9^ CFU/mL 43.1 and 71.3%; marbofloxacin 10^6^ CFU/mL 86.2% and 85.0%, 10^7^ CFU/mL 81.9% and 99.7%, 10^8^ CFU/mL 45.9% and growth, 10^9^ CFU/mL growth and 44.0%; pradofloxacin 10^6^ CFU/mL 98.7% and 100%, 10^7^ CFU/mL 99.6% and 99.99%, 10^8^ CFU/mL 94.4% and 97.9%m 10^9^ CFU/mL 31.7% and 63.7%. At the MSDC, killing by the four fluoroquinolones was more rapid as follows with killing summarized here at 1 and 12 h after drug exposure.: enrofloxacin 10^6^ CFU/mL 99.9% and 100%, 10^7^ CFU/mL 99.9% and 99.99%, 10^8^ CFU/mL 99.8% and 99.99%, 10^9^ CFU/mL 93.8% and 99.7%; danofloxacin 10^6^ CFU/mL 98.4% and 100%, 10^7^ CFU/mL 89.6% and 100%, 10^8^ CFU/mL 91.8% and 99.9%, 10^9^ CFU/mL 66.8% and 83.6%; marbofloxacin 10^6^ CFU/mL 99.7% and 100%, 10^7^ CFU/mL 99.7% and 99.99%, 10^8^ CFU/mL 91.8% and 99.9%, 10^9^ CFU/mL 66.8% and 83.6%; pradofloxacin 10^6^ CFU/mL 100% and 100%, 10^7^ CFU/mL 99.99% and 100%, 10^8^ CFU/mL 99.99% and 100%, 10^9^ CU/mL 99.99% and 100%. Similar trends in in vitro killing were sent for strains of *P. multocida* tested against the same drugs and over the same varying bacterial densities and drug concentrations. As a general observation, as the bacterial density increased, a decline in killing over the exposure times investigated was seen. With *M. haemolytica* strains the reduction in killing seen at higher densities was less pronounced with pradofloxacin versus some of the other quinolones tested. How this relates to pradofloxacin being a dual targeting agent remains to be determined. For example, one could speculate that at the higher bacterial densities, the reduction in killing by some quinolones could be due to selection of first step resistant mutants that spontaneously arise in high density populations. Further investigations will be required to answer this question.

In vitro kill measurements have some limitations, i.e., in vitro drug concentrations remain constant over the duration of the assay whereas in vivo, drug elimination occurs. Additionally, what drug concentration is best to use that represents something close to clinical relevance? Despite limitations, such measurements allow drug-to-drug comparison under controlled experimental conditions thereby allowing important differences between drugs to be defined. For such measurements, bacterial densities ranging from 10^5^ to 10^9^ CFU/mL have been used in kill assays [139,154,164]. The speed of kill and the extent of kill over time can be determined for various Bug–Drug combinations as can categorization of bactericidal or bacteriostatic and killing between agents can be compared statistically [154,164].

### Antimicrobial Data and Clinical Outcome

Considerable debate exists as to what type of antimicrobial data should influence clinical practice. Most investigators would agree that clinical outcome remains the single most important parameter, as it determines whether the patient has a favorable clinical response or not. Measurements of clinical outcome are based on clinical trials in which a new agent, or an existing agent being investigated for a new clinical indication, is compared to an antimicrobial agent currently approved for the investigated indication. In veterinary medicine, a new antibiotic may be compared to placebo or vehicle minus active ingredient. In such trials, clinical outcome is measured at predetermined endpoints, and in some studies bacteriological response or outcome is measured. Previously, antimicrobial clinical trials in human medicine were not universally suited to address antimicrobial resistance concerns for the following reasons:

1. They were powered to show equivalency or non-inferiority; there was no regulatory requirement to design trials to demonstrate superiority.
2. Most—if not all—trials excluded patients with an organism (pathogen) that had reduced susceptibility or resistance to either study drug.
3. Endpoint measurements were often conducted at a time when there was unlikely to be a measurable difference between the compounds—i.e., at a predetermined time after the end of antimicrobial therapy.
4. Depending on the type of trial, microbiological evaluations was lacking or inadequate or restricted to subsets of the study populations.
5. Many trials investigate one dose without dosage adjustments based on patient characteristics (i.e., weight).
6. Patients enrolled in clinical trials were often well defined (i.e., meet inclusion criteria or more importantly, do not have any exclusion-related characteristics); they may not have been designed to accurately reflect the patient population in which resistance was most likely to arise (i.e., the “real world” population).
7. Antimicrobial failure due to resistance may not have been measured.

Paul et al. commented that recruitment of patients with critical priority antimicrobial resistant bacteria has not been successful to date and this needs to be improved moving forward [170]. They further indicate a gap exists in the available evidence on the effects of new antimicrobial agents on infections caused by antimicrobial resistant pathogens.

Some of these same variables may or may not be a concern in veterinary medicine. As stated above, currently and almost uniformly, antibiotics are approved based on the demonstration of non-inferiority of a new drug when compared with a standard antimicrobial agent approved for a specific indication. However, such trials may fail to take into account various microbiological or pharmacological parameters that could be used to determine optimal versus suboptimal dosing. As such, these parameters may not necessarily affect (or they may) clinical outcome but may have a huge impact on the selection of drug-resistant pathogens—and thereby compromise antimicrobial activity over time—a suboptimal outcome for maintaining drug activity against target pathogens over time (i.e., months/years/decades).

As previously stated, clinical trials for antimicrobial agents are generally flawed as the design is to compare the investigational antimicrobial agent to one already approved for the infectious diseases being treated. Such studies have traditionally been powered to show equivalency or non-inferiority and strict exclusion criteria may exclude subjects that are present in real world clinical settings (i.e., infection with a drug-resistant bug). Such investigations give the impression of equivalency between drugs—which may be the case when considering clinical outcome; however, fundamental differences between compounds as determined by MIC, MPC and kill assays may provide evidence for preferential use of a particular compound or drug class in certain clinical situations where selection of drug-resistant organism is a concern. Additionally, such measurements may provide critical information on the possibilities for shorter durations of therapy and or the likelihood for resistance selection/prevention with specific drugs.

## 8. Anti-Inflammatory Properties of Fluoroquinolones

Macrolides are often thought of as having anti-inflammatory properties and compelling evidence exists that they do [165]. However, macrolides are not the only antimicrobial drug class with immunomodulatory properties. Dalhoff and Shalit provided a comprehensive review on the immunomodulatory effects of quinolones [171]. They indicate that, in general, most fluoroquinolones superinduce in vitro interleukin 2 synthesis but inhibit synthesis of interleukin 1 and tumour necrosis factor alpha (TNF-alpha). Additionally, these compounds significantly enhance the synthesis of colony-stimulating factors. Fluoroquinolones attenuate cytokine responses and some agents enhance haematopoiesis by increasing the concentration of colony-stimulating factor in lungs, bone marrow and shaft. They suggest the mechanisms potentially explaining fluoroquinolone immunomodulating effects include (1) some type of effect on intracellular cyclic adenosine 3′,5′ monophosphate and phosphodiesterase, (2) an effect on nuclear factor kappa B (NK-kB), activator protein 1, nuclear factor-interleukin-6, nuclear factor of activated T-cells and (3) triggering effect on the leukocytic equivalent of the bacterial SOS response. Sachse et al. investigated the anti-inflammatory effects of ciprofloxacin in *S. aureus* Newman induced nasal inflammation in an in vitro model using human nasal epithelia cells [172]. Chronic rhinosinusitis may be related to enterotoxins from *S. aureus*. Human nasal epithelial cells had inflammation induced with supernatants from *S. aureus* for 12 h following which the cells were co-incubated with ciprofloxacin, clarithromycin or prednisolone for 12 h and interleukin-8 (IL-8) was quantified following 12 and 24 h. Stimulation resulted in an increase in IL-8 synthesis following 12 h and then decreased over the next 12 h and in the absence of any intervention. Regardless, co-incubation of human nasal epithelial cells with ciprofloxacin or prednisolone resulted in a significant (*p* < 0.05) decrease in IL-8 synthesis. Co-incubation with clarithromycin resulted in a non-significant reduction in IL-8 levels. The authors concluded ciprofloxacin exerts anti-inflammatory effects under the experimental conditions measured. Zusso et al. investigated ciprofloxacin and levofloxacin for attenuation of the microglia inflammatory response using cell cultures from Sprague–Dawley rats [173]. Ciprofloxacin and levofloxacin bound to the MD-2 pocket of the Toll-Like Receptor-4 (TLR_4_)-MD-2 complex thereby preventing lipopolysaccharide (LPS)-introduced secretion of pro-inflammatory cytokines and activation of NK-kB in primary microglia and both compounds diminished binding of LPS to TLR_4_-MD-2 complex. Liu et al. reported levofloxacin activity on rabbit inflammatory fibroblast synoviocytes in vitro [174]. Markers measured included matrix metallopeptidases (MMP) 1 and 3, Tissue inhibitor of metalloproteinases 1 (TIMP-1), cyclooxygenase-2 (COX2), prostaglandin E2, interleukin 6 and caspase -3,-8,-9. Following levofloxacin exposure for 24 h, cell viability decreased (concentration dependent), caspase-3 protein expression levels increased while MMP-3 remained unchanged. The mRNA levels for MMP-1, MMP-3 and TIMP-1 all increased while IL-6 and COX-2 decreased and caspase-3 and -8 increased in a concentration-dependent manner. The authors indicated that the anti-inflammatory and anti-proliferative effects of levofloxacin on synoviocytes are mediated by inhibiting inflammatory cytokines and inducing apoptosis.

Assar and colleagues provided a comprehensive review on the immunomodulatory effects of fluoroquinolone compounds. Their review focused on compounds used in human medicine [175]. They summarize that indirect immunomodulating properties of fluoroquinolones was via suppressing pro-inflammatory cytokines (interleukin 1 and 6), TNF–alpha and super-inducing interleukin 2—which increase growth and activity of T and B lymphocytes. Singh et al. investigated sparfloxacin in goats for immunological effects [176]. Animals (four each) were in three groups: saline control, antigen control, animals exposed to sparfloxacin and sheep red blood cells. Blood was sampled at 1, 7, 10, 14, 21, 28, 35 and 42 days of the experiments. Haemagglutination testing for humoral immune response was measured and no impact was seen; however, delayed-type hypersensitivity—for evaluating the cellular immune response—was significantly (*p* < 0.01) higher in sparfloxacin-treated animals as compared to antigen-treated animals. Absolute lymphocyte count differed significantly (*p* < 0.01) in the sparfloxacin-treated group as compared to the antigen treated group. The authors concluded that sparfloxacin had some immunomodulatory effect with respect to cell-mediated immunity in goats.

Majeed et al. reported on the immunomodulating effects of lomefloxacin in male albino mice. Dosages of 12.5, 25 and 50 mg/kg were investigated. Delayed type hypersensitivity assays showed immunosuppression effects of the higher dosages. A dose-dependent effect was seen in suppression of humoral immune responses in both haemagglutination and mice lethality assays. The authors concluded significant immunomodulating potential for lomefloxacin.

Tsivkovskii and colleagues investigated levofloxacin for immunomodulating properties [177]. The study evaluated high levofloxacin drug concentrations (by aerosol administration) on pro-inflammatory cytokine secretion by immortalized human bronchial epithelial cells in vitro. Levofloxacin showed a dose-related reduction in interleukin 1 (four-fold) and 8 (two-fold) with 300 µg/mL but had not affected mRNA levels or nuclear factor-KB-dependent promotor activity. The authors suggest that high pulmonary levofloxacin concentration—as attainable by aerosol drug delivery—may have additional benefits independent of antimicrobial properties. Zusso et al. investigated fluoroquinolones for attenuating the microglia inflammatory response in primary microglia [173]. Ciprofloxacin and levofloxacin inhibited lipopolysaccharide (LPS)-induced secretion of pro-inflammatory cytokines and activation of NF-kB in primary microglia and reduced binding of LPS to toll-like-receptor 4 and myeloid differentiation proteins-2 complex.

Enrofloxacin (at therapeutic doses) was investigated for the effect on circulating lymphocyte subpopulation in pigs. In comparing pigs given enrofloxacin versus a control group given phosphate-buffed saline, Pomorska-Mol and Pejsak measured lymphocyte subpopulations by fluorochrom-labeled antibodies [178]. Following enrofloxacin treatment, the concentration and percentage of CD8+ cells decreased significantly and the CD4/CD8 ratio increased significantly.

Mukherjee and Dash investigated enrofloxacin in bovine subclinical mastitis [179]. Immunomodulation was measured using myeloperoxidase and acid phosphates enzyme levels in milk leukocytes [179]. From forty-five cows in three groups, (1) healthy controls, (2) cows with subclinical mastitis receiving 150 mg of enrofloxacin and (3) cows with subclinical mastitis receiving sterile phosphate-buffered saline and treatment were intra-mammary with observations recorded up to 30 days post-treatment. Acid phosphate levels increased by 70% on day 3 of enrofloxacin-treated animals compared to 18.7% in the PBS-treated group; myeloperoxidase levels increased 32% versus 18%, respectively. The authors concluded that enrofloxacin use in subclinical mastitis at suboptimal dose reduced bacterial load by increasing bactericidal enzyme levels in milk polymorphonuclear cells thereby indicating immunomodulation in bovine mastitis.

The immunomodulation effects of moxifloxacin on secretions of cytokines by LPS stimulated human monocytes from 10 healthy volunteers was investigated [180]. Moxifloxacin shares many similar properties to pradofloxacin—a veterinary fluoroquinolone. Three, six and twenty-four hours following exposure to moxifloxacin, LPS- or Pansorbin-stimulated monocytes and various interleukins and TNF-alpha were measured. Both LPS- and Pansorbin-stimulated monocytes resulted in a significant increase in cytokine secretions. Moxifloxacin significantly (*p* < 0.01) inhibited secretions of interleukin 1 alpha from LPS-stimulated monocytes from each of the 10 donors; TNF-alpha was significantly (*p* < 0.01) inhibited from monocytes of 6/10 donors. Additionally, there was a non-statistically significant trend toward inhibition of secretion of interleukin 6, 10, 12. Cytokine secretion from monocytes stimulated by Pansorbin was not statistically inhibited by moxifloxacin.

Velho et al. studied the immunomodulatory effects of moxifloxacin on protection in sepsis in mice [181]. Moxifloxacin inhibited the secretion of TNF-alpha and interleukin 1 beta in THB1 cells stimulated with either LPS or *E. coli*. Intraperitoneal administration increased the survival rate of mice with severe sepsis by 80% (*p* < 0.001) and significantly reduced plasma levels of cytokines. The authors suggest the immunomodulating effects of moxifloxacin appear unrelated to either antimicrobial activity or induction of DNA damage.

Yao et al. investigated the regulatory effects of danofloxacin on LPS-induced immune stress [182]. Using a fever piglet model, danofloxacin was shown to significantly suppress plasma and alveolar macrophage 3D4/2 cells levels of interleukin 1 beta, TNF-alpha, interleukin 6, nitric oxide and prostaglandin E2 when compared to the LPS challenged group.

Fajt et al. measured the effects of danofloxacin (and tilmicosin) on neutrophil function and lung consolidation in *M. haemolytica*-induced pneumonia in beef heifer calves [183]. Treatment was at 20 h after challenge. Peripheral blood neutrophils were collected at 3, 24 and 48 h after treatment. Neutrophil function assays included random migrations under agar, cytochrome reductions, iodination, *S. aureus* ingestion, chromotoxin and antibody-dependent and independent cell-mediated cytotoxicity. Apoptosis was also measured. Comparisons were made between danofloxacin, tilmicosin and saline-treated animals. Significant differences from any of the neutrophil function assays or neutrophil apoptosis was not seen between the groups. There were no differences in lung consolidation amongst the treatment groups. The authors concluded that neither danofloxacin or tilmicosin had any clinically significant effects on neutrophil function or apoptosis.

Endo and colleagues reported on marbofloxacin and its impact on fever and blood properties in 48 healthy thoroughbreds transported over a long distance [184]. Horses were premedicated with interferon-alpha for 2 days prior to transport. Horses were randomly assigned to receive either enrofloxacin (5 mg/kg once), marbofloxacin (2 mg/kg once) or saline (0.9% NaCl, 10 m (IV once) ≤1 h prior to transport. Each group contained eight males and eight females with a transportation time of ~26 h and a distance of 1210 km with clinical and haematological examination prior to and after travel. Neutrophil to lymphocyte ratios were significantly lower in marbofloxacin-treated animals and serum amyloid A levels were significantly lower in horses receiving either quinolone as compared to the control group. Post-transport treatments were lower in both drug groups versus control horses.

Yudhawati and Wicaksono reviewed the immunomodulatory effects of fluoroquinolones in community-acquired pneumonia-associated acute respiratory distress syndrome [185]. They indicated the immunomodulatory effects of quinolones are mostly anti-inflammatory but the precise mechanisms are not yet fully elucidated. They argue that in community-acquired pneumonia, there is an excessive pro-inflammatory response. The immunopathogenesis of pneumonia is in part due to infiltration of innate immune cells, release of inflammatory mediators and injury pathways facilitating lung damage. Pulmonary edema contributes to respiratory distress syndrome.

In summary, it appears clear that fluoroquinolones have systemic effects (immunomodulatory) in the host that extend beyond antimicrobial properties.

## 9. Endotoxin and Fluoroquinolones

Endotoxin is a lipopolysaccharide (LPS) and is the outermost component of the cell envelope of Gram-negative bacilli. LPS consists of three parts: (1) the O-antigen, which consists of polysaccharide and forms the outer core, (2) an inner core oligosaccharide and (3) Lipid A. All three components are covalently linked and Lipid A is associated with endotoxin toxicity.

Galanos and Freudenberg stated endotoxins are agents of pathogenicity of Gram-negative organisms and are implicated in Gram-negative shock, which is mediated via TNL-alpha and macrophages [186]. Numerous biological effects of LPS and free Lipid A have been described and include leucopenia, leucocytosis, bone marrow necrosis, compliment activation, blood pressure depression, platelet aggregation, induction of plasminogens activator, induction of nonspecific resistance to infection, macrophage activation, induction of colony stimulating factor, prostaglandin synthesis and interferon production, plus many other additional effects.

Sheehan et al. commented on endotoxin (and exotoxin) in intensive care medicine [187]. They indicate that LPS is eliciting sustainable antibody responses and LPS is not readily denatured with heat. Additionally, endotoxins are released at a low but constant rate from viable bacteria but released at substantially higher concentrations during bacterial cell lysis. Lipid A is complex and composed of a disaccharide, myristic acid and species-specific fatty acids related to the bacterial species. Patients are at risk for repeat endotoxin-mediated shock due to lack of adaptive immunity. Functionally, LPS and LPS-binding proteins form complexes on CD14 cells and TLR-4 on the surface of CD4 cells. This in turn activates the intracellular nuclear factor (NF) kappa-beta whose function is regulation of cytokine gene transcription which includes TNF-alpha and interleukin 1. An unfortunate consequence of high LPS concentrations is the unregulated release of cytokines (cytokine storm) and compliment activation given way to septic shock and multi-organ system failure [188,189]. Jarczak and Nierhaus wrote that human innate and adaptive immunity is based on effector cells producing cytokines such as interferon, chemokines and other mediators [190]. An equilibrium of pro- and anti-inflammatory effects is based on complex regulating processes, which, when out of homeostasis can lead to a massive release of cytokines, which has been referred to as a cytokine storm [190]. Unfortunately, in such patients, this may ultimately lead to systemic damage, multi-organ failure or death.

Clinically, patients with Gram-negative septic shock may have symptoms including fever, tachycardia, increased ventilator support, hypotension and poor organ perfusion, neutrophilia, increased procalcitonin and C-reactive protein and organ failure.

Holzheimer reviewed antibiotic-induced endotoxins release and clinical sepsis. Variables impacting antibiotic-induced endotoxin release included the type and location of the infection, strain virulence, Gram stain, mode of application and dosage of antibiotic [191]. Additionally, Holzheimer indicated that different antibiotics may induce release of different forms of endotoxin and a combination of an antimicrobial with inhibition of endotoxin and/or the pro-inflammatory response may decrease endotoxin release and impact mortality.

Skorup et al. investigated if the mode of bacterial killing affected the inflammatory response and associated organ dysfunction [192]. They used a porcine *E. coli* sepsis model. The study involved twenty-six healthy pigs in four groups; (1) bacteria killed by cefuroxime, (2) live bacteria, (3) bacteria killed by heat and (4) bacteria killed by a combination of cefuroxime plus tobramycin. Variables measured included plasma endotoxins, TNF-alpha, interleukins 6 and 10, leukocytes and organ function. Variables were measured at the start of the experiments and then hourly for 6 h. Bacteria with pre-exposure to cefuroxime yielded higher cytokine values for each of the three cytokines than did live bacteria (*p* < 0.05) or pre-exposed to heat bacteria (*p* < 0.01). The addition of tobramycin to cefuroxime significantly reduced the cefuroxime induced cytokines response (*p* < 0.001). The authors concluded that a penicillin-binding protein targeting antibiotic induced increased inflammatory responses that may be associated with organ and cellular function deterioration.

Sjolin and colleagues investigated endotoxin release from *E. coli* after exposure to the aminoglycoside tobramycin, cefuroxime and both drugs in combination [193]. In this interesting study, a clinical isolate of *E. coli* in logarithmic growth phase was exposed to 0.1, 2, 10 and 50 × the MIC of cefuroxime, tobramycin and both drugs in combination. Viable organism counts and endotoxin analysis were determined immediately before and after antibiotic exposure and at 1, 2, 4, 6 and 24 h after drug exposure. Liberation of endotoxin was proportional to the number of killed bacteria. Over all concentrations tested, the highest release of endotoxin per killed bacteria was for cefuroxime, lower for tobramycin and lowest for the combination of cefuroxime/tobramycin (*p* < 0.001). Increasing tobramycin concentrations lead to a reduction in endotoxin release.

Shalit et al. compared the immunomodulatory effects of ciprofloxacin and moxifloxacin and granulocyte colony-stimulating factor (G-CSF) by studying the repopulation of hematopoietic organs and cytokine production in leukopenic mice following cyclophosphamide administration [194]. At 4 days post cyclophosphamide treatment, severe neutropenia was seen. The quinolone and G-CSF treated animals had white blood cells >500/µL at 4 days as compared to 50% of saline treated mice and a 1.4–4.3-fold increase in myeloid progenitors in bone marrow but not in the spleen. Quinolone therapy significantly enhanced colony-stimulating activity in the bone shaft, spleen, lung and bladder on different days. Cyclophosphamide administration resulted in a 22.5–93-fold decrease in GM-CSF and lL-6 level whereas treatment with quinolones resulted in a 2–4-fold increase in GM–CSF and no effect on iL–6 production. The authors concluded moxifloxacin and ciprofloxacin given to cyclophosphamide-injected mice revert some of the immunosuppressive effects of cyclophosphamide.

Nau and Eiffert reviewed strategies for minimizing pro-inflammatory and toxic bacteria products in hosts and approaches to improving life-threatening infections [195]. They note bacterial components such as endotoxin, teichoic and lipoteichoic acids, peptidoglycans and DNA can induce or enhance inflammatory mediators and responses. They further present that antimicrobial agents inhibiting bacterial protein synthesis induce smaller quantities of toxic compounds or pro-inflammatory mediators (in vitro and in vivo) than does killing by beta-lactam or other cell wall active agents. Interestingly, they indicate that higher drug concentrations liberate less-toxic products or pro-inflammatory mediators than do drug concentrations close to the MIC. In various animal models, a low release of pro-inflammatory bacterial compounds was associated with reduced mortality. When treatment with a beta-lactam drug involved pre-treatment with a protein synthesis inhibitor, a reduction in the strong release of bacterial products was seen.

## 10. Drug Classes and Impact on Pro-Inflammatory Bacterial Compounds

### 10.1. Beta-Lactam Drugs

Mellado et al. showed *Neisseria meningitidis* exposed for 1–2 h to penicillin released more endotoxin than did *N. meningitidis* during uninhibited growth but with longer observations, spontaneous release during bacterial replication was higher than with penicillin or ceftriaxone exposure [196]. As summarized by Nau and Eiffert, in the presence of beta-lactam antibiotics, the following have been reported to occur [195]: rapid cell lysis due to inhibition of penicillin binding protein (PBP) 1A and 1B; occurrence of osmotically sensitive cell-wall-deficient round cells due to inhibition of PBP-2; filament formation due to inhibition of PBP-3 which leads to strong endotoxin production and release.

Investigations with the beta-lactam antibiotics aztreonam and cefuroxime—which bind to penicillin binding protein–3—were found to release larger quantities of free endotoxin as compared to the PBP-2-binding antibiotic imipenem [197,198].

### 10.2. Aminoglycosides

Shenep et al. investigated chloramphenicol, gentamicin and moxalactam in a rabbit *E. coli* sepsis model [199]. They measured viable bacteria, free endotoxin and total endotoxin in blood samples. Chloramphenicol did not appear to induce endotoxin liberation. Free endotoxin levels increased and bacteremia declined following treatment with gentamicin or moxalactam; however, free endotoxin was as much as 20-fold higher in moxalactam-treated animals (*p* < 0.05) as compared to paired rabbits given gentamicin. Kadurugamuwa and Beveridge investigated *Pseudomonas aeruginosa* and gentamicin [200]. They indicate that *P. aeruginosa* (and other Gram-negative bacteria) liberate during normal growth membrane vesicles containing endotoxin, outer membrane proteins, protease, alkaline phosphatase, phospholipase C and peptidoglycan hydrolase. Alkaline phosphatase along with pro-elastase contribute to periplasmic component packaging. Liberated vesicles fuse with epithelial cell membranes and can thereby release virulence factors into host cells and degrade cellular components contributing to infection. Aminoglycosides may interfere with the packing order of lipids and destabilize bilayer membranes, which can result in cell lysis. Gentamicin increased the release of membrane vesicles 3–5 fold and this could account for bacteria-mediated toxicity in patients treated with an aminoglycoside.

### 10.3. Fluroquinolones

Exposure of *E. coli* to ciprofloxacin for 60 min showed a 3 log_10_ reduction in colony-forming units but not an equivalent decrease in bacterial numbers by microscopy/flow cytometry, thereby suggesting organisms were unable to replicate but were not lysed [201]. Evans and Pollack measured the effect of antibiotic class and drug concentration on the release of LPS from *E. coli*. Release of radiolabeled LPS from *E. coli* 0111:B4 was determined with drug concentrations ranging from 0.0625 to 512 µg/mL. The mean values of LPS released depending on the drug were as follows: polymyxin B 40.6%, gentamicin 58.2%, ciprofloxacin 65.8%, ceftazidime 73.1%, tetracycline 75.3% and imipenem 79.7%. Considering time after drug exposure (e.g., 1 h), ceftazidime released 61.9%, imipenem 51.1% and tetracycline 39.7% as compared to polymyxin B 13.5%, gentamicin 9.8% and ciprofloxacin 12.7%. The authors concluded the amount and rate of LPS release from *E. coli* was dependent on antibiotic drug class and drug concentration.

Non-human primates, horses, cattle, pigs, dogs, cats and birds are susceptible to the effects of endotoxin. As such endotoxin is important in veterinary medicine as well.

Gogos et al. compared ciprofloxacin and ceftazidime on cytokine production in patients with Gram-negative bacteria-induced severe sepsis [202]. A total of 58 patients with severe sepsis were studied. Pro-inflammatory cytokines measured included TNF-alpha, interleukin-1b, IL-6, IL-8 and the anti-inflammatory cytokine IL-10. Measurements were taken at baseline and at 24 and 48 h after the first dose of antimicrobial agent. Mortality rates were similar between treatment groups; however, serum TNF-alpha and IL-6 levels at 24 and 48 h were significantly lower in the ciprofloxacin group whereas the IL-10/TNF-alpha ratio was significantly higher when compared to the ceftazidime group. The author concluded ciprofloxacin may have immunomodulating effects on septic patients by attenuating the pro-inflammatory response.

Purswani and colleagues studied the effects of ciprofloxacin on lethal and sublethal challenge with endotoxin and on cytokine responses in a mouse model [203]. Mice were injected intraperitoneally with LPS ranging from 200 to 1000 µg. Mice were pretreated with 0.0–6.0 mg of ciprofloxacin ~1 h before the LPS challenge. Cytokine responses were measured from blood after 72 h. Mice were consistently protected with 225–300 mg/kg of ciprofloxacin given 1 h before the LPS challenge (*p* < 0.0001). Additionally, ciprofloxacin significantly attenuated the production of TNF-alpha and IL-12 responses. The authors indicate that ciprofloxacin may prevent endotoxin-mediated deaths and attenuate early cytokine responses in hosts.

Prins et al., in a mini review, summarized data on the clinical relevance of antibiotic-induced endotoxin release [204]. The authors acknowledge that antibiotic therapy liberate endotoxin but appropriate antimicrobial therapy reduces mortality [205]. As to the potential differences between antibiotic drug classes and/or individual agents and the liberation of endotoxin, the authors acknowledge that most studies have been done in vitro and many variables may affect extrapolation of such observations to clinical settings.

Trautmann et al. measured endotoxin release due to ciprofloxacin using three different measurements [206]: (1) Limulus amaebocyte lysate test, (2) ELISA-based method based on capture of LPS by monoclonal antibodies and (3) indirect by measuring the ability of antibiotic-induced LPS to impact TNF-alpha release from a monocytic cell line. The Limulus and ELISA assays showed a low endotoxin release by ciprofloxacin and the LPS had bioactivity of TNF-alpha induction. The authors indicate a critical role of methods in measuring antibiotic-induced LPS release.

Khan et al. investigated three fluoroquinolones (trovafloxacin 100 mg/kg, ciprofloxacin 250 mg/kg, tosufloxacin 100 mg/kgs) for protection against LPS-induced death in a mouse model [207]. Mice were injected with a lethal dose of LPS. All agents significantly (*p* = 0.002, *p* = 0.0001) protected mice from death and reduced serum levels of IL-6 and TNF-alpha in LPS-treated mice. Specifically, trovafloxacin resulted in significant reductions in IL-6 (*p* = 0.009) and TNF-alpha (*p* = 0.01) 1 h after LPS injection; similar results were seen for ciprofloxacin in IL-6 (*p* = 0.02) and TNF-alpha (*p* = 0.05).

Elmas et al. investigated if *E. coli* endotoxin-induced endotoxemia affected the deposition of enrofloxacin in rabbits [208]. While the value of distribution at steady state was significantly lower (*p* < 0.05), the elimination half-life of enrofloxacin was not affected by LPS administration. Enrofloxacin treatment of *E. coli* bovine mastitis was found to be beneficial due to its bactericidal activity and was not associated with enhanced resorption of endotoxins [209]. In swine administered with a single IV dose of enrofloxacin (5 mg/kg) and LPS (2.0 microg/kg), decreased clearance of enrofloxacin was seen and the terminal elimination half-life significantly increased. LPS challenge did not affect urinary excretion of enrofloxacin but did increase excretion of ciprofloxacin (enrofloxacin metabolite) [210].

## 11. Fluoroquinolones

### 11.1. Ciprofloxacin

Ciprofloxacin tablets were approved for clinical use in humans in October 1987; the intravenous formulation was introduced in 1991. Ciprofloxacin was seen as a broad-spectrum agent that was widely distributed in the body (less in the central nervous system) and an oral agent for treatment of susceptible strains of *Pseudomonas aeruginosa*. There are numerous clinical indications in humans for ciprofloxacin therapy. In both food and companion animals, ciprofloxacin is a metabolite of enrofloxacin and it is in that context that it will be discussed further. While ciprofloxacin is used in companion animals, it is an off-label usage as it is not approved for use in veterinary medicine.

### 11.2. Danofloxacin

Bovine respiratory disease (BRD) (pneumonia) is an acute pulmonary infectious process affecting cattle with significant morbidity and mortality exists. Peek (2005) suggests that infectious causes in cattle (calves and adult) represent the most commonly encountered category in first opinion practice and may be individual or group based complaints [211]. Apley [212] indicated there were a number of basic steps required for successful therapy including: (1) identification of disease challenges /probable pathogens, (2) a case definition describing exactly how to select animals for therapy, (3) described treatment regimens including (dose, route, duration, frequency, injection site, volume per site and slaughter withdrawal times), (4) case definition for success or failure, (5) descriptions of second/possibly third treatment regimens and (6) consistent application of treatment regimens to allow for comparative evaluation.

For BRD disease complex [213], viral infection along with numerous potential stressors (environmental, nutritional, management and infectious) predispose animals to secondary bacterial infections necessitating antibacterial treatment. The principal bacterial pathogens associated with BRD include *Mannheimia haemolytica*, *Pasteurella multocida* and *Histophilus* (formerly *Haemophilus*) *somni*. Atypical organisms such as *Mycoplasma* spp. [214] and *Chlamydophila* spp. [215] also contribute to BRD complex. Timely, optimal, effective therapy is needed to impact morbidity, mortality and reduce the likelihood for resistance selection with antibacterial exposure. Arguably, optimal antimicrobial therapy includes an antimicrobial spectrum to include both typical and atypical pathogens and minimized resistant mutant subpopulations selection during drug exposure (discussed under the MPC section). Numerous antibacterial drugs have been approved to treat BRD and include ceftiofur, florfenicol, oxytetracycline, penicillin, tilmicosin, tulathromycin and the fluoroquinolones danofloxacin, enrofloxacin, marbofloxacin and most recently pradofloxacin (approved 2024).

Danofloxacin mesulylate is a synthetic fluoroquinolone for the treatment/control of bovine respiratory disease caused by *M. haemolytica* and *P. multocida*. The drug is administered subcutaneously at a dose of 8 mg/kg BW as a one-time injection. From isolates collected between 1996 and 1997, MIC_50_ and MIC_90_ values for 106 *M. haemolytica* strains were 0.06 and 0.06 µg/mL as compared to ≤0.015 and 0.06 µg/mL for 94 *P. multocida* strains. For strains collected in 2013, MIC_50_ and MIC_90_ values for 507 *M. haemolytica* strains were 0.03 and 0.06 µg/mL as compared to ≤0.008 and 0.12 µg/mL for 324 *P. multocida* strains.

Parker–Graham et al. investigated the pharmacokinetics of danofloxacin following a single intramuscular injection (1 mg/kg) in koi (*Cyprinus carpio*) [216]. A total of sixty-nine healthy fish were used in eleven treatment groups with six fish/group. Three fish were untreated controls. Injection was in the left epaxial musculature at 15, 30 and 45 min and 1, 4, 12, 24, 72, 96, 120 and 144 h after drug administration, fish were euthanized and blood, liver, spleen, gill, kidney (anterior and posterior), skin, muscle and scales were collected. The C_max_ was 8.31 µg/mL after ~45 min and the elimination of half-life was 15 h. Danofloxacin was detected in cells/tissues in 6/6 fish euthanized 15 min after drug administration and from 3/6 fish euthanized after 144 h.

The PK/PD of danofloxacin in experimentally induced pneumonia with *M. haemolytica* in calves was reported by Sarasola et al. [217]. A total of 33 male Friesen calves, 11–13 weeks of age and weighing 65.5–106 kgs were used and experimentally infected by endo-bronchial deposition (10 cm into principal bronchus) of 300 mL of inoculum (~10^8^ cfu/mL—acceptable range 5 × 10^7^ to 5 × 10^8^ cfu/mL) by a fiber-optic endoscope. There were three treatment groups with eleven animals per group: (1) danofloxacin (6 mg/mL) administered as a single IV bolus injection at 0.738 mg/kg; (2) danofloxacin (0.75 mg/mL) administered as a continuous IV infusion over 36 h to give a total infused dose of 0.645 mg/kg—the total dose being 0.738 mg/kg following small IV boluses at 0.93 mg/kg; and (3) saline at 0.123 mg/kg as a single IV bolus injection to match the dose volume equivalent. Clinical signs monitored included respiratory rate, rectal temperature, character of respiration and general demeanor and were collected at times 0, 4, 8, 12, 24, 24, 36 and 48 h thereafter. Blood and bronchial specimens were collected at various timed intervals. Tissue samples were collected from euthanized animals. The single bolus danofloxacin infusion was more effective than the continuous infusion dose as evidenced by a significantly lower (*p* < 0.05) number of cows with *M. haemolytica* in bronchial secretions after treatment. Additionally, lower rectal temperatures were seen in the first 24 h after treatment initiation. The authors concluded that for cattle with *M. haemolytica* induced respiratory disease, danofloxacin exhibited concentration-dependent antimicrobial activity.

A summary of select clinical trials is provided in Table 4. Rowan et al. investigated the efficacy of danofloxacin (6 mg/kg) in cattle in Europe with respiratory disease [218]. A total of 128 animals treated with danofloxacin completed the study. *M. haemolytica*, *P. multocida* and *H. somnus* were found in animals across farms in France, Ireland and the United Kingdom. At day 2, 54% of danofloxacin-treated calves met the criteria for retreatment as compared to 72% for tilmicosin (*p* < 0.01). Danofloxacin-treated animals had a rapid reduction in mean rectal temperature by day 1 (*p* < 0.05 compared to tilmicosin treated animals) and there was a rapid reduction in the prevalence and severity of clinical signs (i.e., abnormal respiration/depression) on days 4 and 10 compared to day 0. Danofloxacin was clinically efficacious in cattle treated for naturally occurring bovine respiratory disease.

Sunderland et al. investigated danofloxacin (6 mg/kg) for treatment of young calves with *E. coli* associated diarrhea on European farms [220]. A total of 267 animals were treated with danofloxacin (98 received gentamicin; 37 received baquiloprim/sulphadimidine). *E. coli* was isolated from 90 to 100% of calves pre-treatment; the prevalence of serotypes K99 and F41 ranged from 8 to 46% and 46 to 92% respectively. In both treatment groups, 92.9–93% of calves showed clinical improvement. For both treatments, significant reduction in the severity of clinical signs was seen on days 4 and 10 (compared to day 0 *p* < 0.05). There were no significant differences between treatments. It was concluded that danofloxacin was safe and efficacious for treatment of *E. coli*-associated diarrhea in calves.

Caproni and colleagues reported on the clinical efficacy of danofloxacin (6 mg/kg) for naturally occurring infectious diseases (respiratory disease, enteritis, metritis, omphalitis) in cattle in Brazil [219]. A four-point scoring was used for disease severity with 0 = normal, 1 = mild, 2 = moderate and 3 = severe and animals with scores of 0 and 3 were excluded from the study. On day 0, animals scoring 1–2 received danofloxacin (6 mg/kg) by subcutaneous injection. On day 2 cured animals (score 0) were removed from the study. Non-responding animals received a second danofloxacin injection. The study concluded on day 4 with final animal examinations. Specimens (fecal, nasal, vagina, navel) were collected prior to initiation of treatment on day 0. In total, 1019 cattle were treated of which 84.1% (857) were cured (52.4% following one dose and 31.7% following two doses). In considering individual clinical conditions, response rates were as follows: respiratory disease 79.8%, enteritis 92.6%, metritis 76.5% and omphalitis 88.0%. A total of 805 bacterial isolates were tested for susceptibility to danofloxacin of which 70.4% were considered susceptible, 9.6% resistant and the remainder intermediate. Considering strains of *E. coli*, 219/303 (72.3%) were fully susceptible as compared to 25/32 (78.1%) for *K. oxytoca*, 57/77 (74.0%) for *K. pneumoniae*, 49/61 (80.3%) for *P. mirabilis*, 93/115 (80.9%) for various species of Staphylococci and 53/108 (49.1%) of various species of Streptococci. A total of 24/32 (75.0%) was fully susceptible to danofloxacin. Danofloxacin was concluded to be safe and efficacious for treating the naturally occurring infections investigated in cattle.

### 11.3. Enrofloxacin

Enrofloxacin was first approved for use in cattle in 1998, in dogs and cats in 1989 and in swine in 2008. It has broad spectrum activity agent Gram-negative and Gram-positive bacteria along with activity against atypical microorganisms. Enrofloxacin is a unique molecule as its main metabolite is ciprofloxacin—a metabolite that is an antimicrobial agent with broad spectrum activity. The antibacterial effects of enrofloxacin and the ciprofloxacin metabolite are additive [75,234]. Following oral administration, enrofloxacin is close to completely absorbed with protein binding ranging between 30 and 46% depending on species. The drug is widely distributed to various body fluids and tissue (Table 1). The drug is metabolized by the liver (to ciprofloxacin) with renal excretion. In considering the conversion of enrofloxacin to ciprofloxacin, Trouchon and Lefebvre in summarizing data from numerous publications indicated the percentage of ciprofloxacin from enrofloxacin metabolism in different animals: dogs 40%, dairy cows 59%, steers 64%, chickens <10%, pigs 51% and goats 34% [1].

As previously indicated, ciprofloxacin is widely used in human medicine. This single observation is important when considering MIC and MPC data. Richez et al. reported ciprofloxacin accounted for approximately 25% of total drug concentrations, based on AUC, in calves and adult cattle injected with enrofloxacin (5 mg/kg) [235]. After subcutaneous injection, ciprofloxacin plasma concentrations peaked at 1.68 and 2.28 µg/mL (~5 hours). The C_max_ for enrofloxacin was 0.733 µg/mL. Stegemann et al. reported the plasma pharmacokinetics of enrofloxacin administered to cattle (7.5 mg/kg) [236]. The C_max_ was reported to be 0.83 µg/mL (HPLC) to 1.71 µg/mL (bioassay), and the AUC was 7.8 µg/mL (HPLC) to 18.9 µg/mL (bioassay). Stegemann et al. suggested when calculating C_max_/MIC and AUC/MIC ratios HPLC derived data greatly underestimates the drug potential by not considering active metabolites such as ciprofloxacin. As such, the combined antimicrobial effects of enrofloxacin/ciprofloxacin need to be considered to understand the total antimicrobial effect.

McKeller et al. reported the pharmacokinetics of enrofloxacin (2.5 mg/kg) and danofloxacin (1.25 mg/kg) in plasma, inflammatory exudate and bronchial secretions of calves following subcutaneous administration [115]. AUC values for enrofloxacin in plasma and exudate were 22–24% higher using a bioassay than by HPLC. With danofloxacin, AUC values were higher by bioassay (than by HPLC) for exudate (36%) but lower for plasma (16%)—the reason(s) for this discrepancy were unclear. AUC values (HPLC analysis) for enrofloxacin (includes metabolite ciprofloxacin) in serum and exudate were 2.5 µg/mL and ~3.3 µg/mL versus ~1.6 µg/mL and 2.5 µg/mL respectively—values 52% and 32% higher respectively for enrofloxacin. Comparative C_max_ values (HPLC analysis) for enrofloxacin in plasma and in exudate were ~0.34 and ~0.27 versus ~0.23 and ~0.19 respectively for danofloxacin—values 48% and 39% higher for enrofloxacin respectively. For enrofloxacin, AUC/MIC_90_ ratios in plasma against *Pasteurella* spp., *M. bovis* and *H. somnus* were 167, 10 and 84 respectively and for danofloxacin, 110, 3 and 27 respectively. AUC/MIC_90_ values in exudate for enrofloxacin respectively were 222, 13, 111; for danofloxacin 167, 5, 42 respectively. Comparing C_max_/MIC_90_ values in plasma for the above-noted pathogens for enrofloxacin, values respectively were 23, 1, 11; for danofloxacin respectively were 15, 0.5 and 4. For C_max_, values in exudate for enrofloxacin were 18, 1, 9 and for danofloxacin 13, 0.4 and 3 respectively.

Davis and colleagues determined the pharmacokinetics and tissue distribution of enrofloxacin and the metabolite ciprofloxacin in calves [75]. Ultra filtration probes were placed in subcutaneous tissue, gluteal musculature and pleural space of five calves. Enrofloxacin was administered subcutaneously dosed at 12.5 mg/kg following placement. Plasma and interstitial fluid samples were collected 48 h after drug administration. Plasma protein binding of enrofloxacin and ciprofloxacin were determined by micro-centrifugation. The mean plasma half-life was 6.8 and 7.3 h respectively for enrofloxacin and ciprofloxacin. The combined peak plasma concentration of both drugs was 1.52 µg/mL and the combined area under the drug concentration curve was 25.33 µg/mL. The free plasma drug concentrations were 54% and 81% respectively for enrofloxacin and ciprofloxacin but higher in tissue fluid.

Blondeau et al. used a unique approach to investigating the in vitro killing of *E. coli*, *S. pseudintermedius* and *P. aeruginosa* strains with enrofloxacin and ciprofloxacin in various ratios (i.e., 50:50, 60:40, 70:30) designed to mimic enrofloxacin and ciprofloxacin percentages in dogs and cats [234]. The percentages in dogs and cats approximate 60–70% enrofloxacin and 30–40% ciprofloxacin. The drug concentration used in the kill studies were 2.1 and 4.1 µg/mL. For *E. coli* a 1.7–2.5 log_10_ (94–99% kill) reduction in viable cells was seen following 20 min of drug exposure. For *S. pseudintermedius*, a 0.89–1.7 log_10_ (92–99% kill) was seen following 180 min of drug exposure and for *P. aeruginosa*, a 0.85–3.4 log_10_ (98–99% kill) following 15 min of drug exposure. The in vitro killing of *S. pseudintermedius* was enhanced with enrofloxacin and killing of *P. aeruginosa* was enhanced with ciprofloxacin. The data suggests the combined presence of enrofloxacin and ciprofloxacin enhances the spectrum of bacterial killing by the drugs in combination.

Wanke et al. investigated enrofloxacin for treatment of canine brucellosis in a dog kennel [237]. A total of twelve dogs (two males, ten females) infected with *B. canis* were administered 5 mg/kg enrofloxacin orally bid for 30 days with females receiving additional dosages during the estral and luteal phases. Females were repeatedly bred by infected males. Serological follow up was carried out for 38 months. Over 14 months from the beginning of the study, all dogs were negative by rapid slide agglutination test. No abortions were seen and all mated female dogs gave birth to healthy puppies. Post-partum vaginal discharges were negative by culture for *B. canis*.

Acute bovine mastitis is an acute inflammatory bacterial infection of the cow’s mammary. Symptoms may include redness, swelling, heat, udder pain and grossly abnormal milk. Fever and lethargy may also be present. Shinozuka and colleagues in a randomized clinical trial tested the effectiveness of enrofloxacin as a second line antibiotic for treating acute mastitis caused by *E. coli* [238]. A total of 42 cows with naturally occurring mastitis were enrolled. Cows (n = 32) were administered oxytetracycline or kanamycin (n = 10) on day 0 with the following observations: ten cows improved, thirty-two cows worsened on day 1 and two of them were found dead. Of the 30 surviving cows, 19 were assigned to receive enrofloxacin and 11 were treated with other antibiotics. Treatment response was measured on days 0 (treatment day) to 3, quarter milk recovery and 60 day survival rate. No differences were seen between treatment groups, which lead the authors to conclude that the use of enrofloxacin as a second line antibiotic for treating *E. coli* acute mastitis could induce a rapid appetite recovery.

Footrot is a painful hoof infection of sheep and endemic in several countries including India, Australia, United Kingdom and New Zealand. Symptoms include lameness, foul smell and separation of the hoof from tissue. Footrot is caused by *Dichelobacter nodosus* [239] and may also be facilitated by *Fusobacterium necrophorum*. Kaler et al. compared parenterally administered oxytetracycline (200 mg/mL; 1 mL/kg IM) or enrofloxacin (7.5 mg/kg IM) in 62 sheep with acute footrot and 30 sheep with chronic footrot [240]. There was a significant correlation (*p* < 0.05) in recovery from lameness and the presence of healing lesions in sheep with acute footrot. A significant difference was seen in the duration of lameness before treatment (median 75 days) and after treatment (median 17 day *p* < 0.01) in sheep with chronic footrot. There were no significant differences between parenteral antimicrobial agents in animals with acute footrot and a median recovery of 7 days. Interestingly, there was a significant difference between topical application of potassium permanganate and the parenteral treatments (*p* < 0.001) in sheep with acute footrot. Kaler *et al* concluded the use of parenteral antibacterials for treating sheep with acute or chronic footrot is highly effective and may offer economic benefits to sheep farmers.

Gutierrez et al. reported on topical (0.5% alginate gel tid) plus oral (10 mg/kg day capsules) treatment with enrofloxacin—hydrochloride-dihydrate for unresponsive deep canine pyoderma [223]. A total of 55 cases (32 severe, 23 very severe) were successfully treated over a 1-year study with a mean day of treatment of 8 days for severe cases and 12 days for very severe cases. Complete success without recurrences were noted at a 2 month follow up. The authors suggest the theoretical high drug concentrations in lesions may explain the study findings.

Outbreaks of acute *A. pleuropneumoniae* in pigs require rapid and effective antimicrobial therapy. Grandemange et al. compared a single injectable dose of marbofloxacin (8 mg/kg) to one or two doses of enrofloxacin (7.5 mg/kg) for treatment of *A. pleuropneumoniae* infections in fattening pigs in Europe [241]. This was a controlled, randomized block, blinded multicenter field study involving four farms. Results were similar for intent to treat (n = 242 pigs) and per protocol (n = 239 pigs) animals. Animals improved rapidly following treatment and by day 7 were clinically well or mild symptoms. Animals cured were 81.8% for marbofloxacin and 81.4% for enrofloxacin on day 7: 84.2% and 82.2% on day 21 respectively. The authors reported marbofloxacin was not inferior to enrofloxacin in this trial and both drugs were safe.

Urinary tract infection is a common infection in dogs. Guidelines for the treatment of acute uncomplicated urinary tract infections have been published [63,64]. Colakoglul and colleagues reported on the efficacy of single dose ceftriaxone to multiple dosing of enrofloxacin for uncomplicated urinary tract infections in dogs [242]. Client-owned non- pregnant (n = 47) dogs with signs of a lower urinary tract infection were enrolled. The study was a prospective, controlled, randomized, blinded clinical trial. Dogs were randomized to receive enrofloxacin (n = 23, 5 mg/kg for 14 days) or ceftriaxone (n = 20, 25 mg/kg IV once). Clinical signs disappeared from 4 to 9 days in enrofloxacin-treated dogs versus 15 days in those receiving ceftriaxone with clinical signs, which improved faster in ceftriaxone treated animals (*p* < 0.0001). Despite these findings, the authors indicate that ceftriaxone is a drug of last resort that limits its use in veterinary clinical practice.

Naval or umbilical infections (omphalitis) in calves occur following the entry of bacteria through the umbilical cord stump following birth. The frequency of naval infections ranges from 3.8 to 28.7% [243]. Common bacteria associated with infection include *S. aureus*, *Streptococcus* spp. and *Clostridium tetani*. Gutierrez et al. measured the effectiveness of enrofloxacin-alginate gel to prevent naval infections in newborn calves [244]. A total of 414 newborn calves were divided into low, medium and high-risk groups for developing omphalitis or omphalophlebitis. Treatments were applied to stumps by “stump-dipping” daily for 3 days with either iodine-polyvinylpyrrolidone (n = 205) or enrofloxacin (0.5%) alginate gel (n = 209). From enrofloxacin-treated animals, one death occurred (unrelated to treatment) and six cases of inconsequential stump fibrosis. In the iodine-treated group, 44 animals developed cord infections and scored as treatment failures (13 high risk, 11 medium risk, 20 low risk; *p* < 0.0435 in the three risk grades). Umbilical stump involution was evident on day 1 in enrofloxacin-treated calves versus 72 h in iodine-treated animals; stump detachment occurred on an average of 30 days versus 32 days (*p* < 0.05) respectively. The authors concluded that alginate gel containing 0.5% enrofloxacin was a successful preventative treatment for omphalitis in newborn calves and rapid umbilical stump involution.

### 11.4. Marbofloxacin

Marbofloxacin is a synthetic broad spectrum fluoroquinolone antibacterial agent for use in North America for dogs and cats. It is water soluble but its solubility decreases in alkaline environments. It is rapidly and nearly completely absorbed from the gut from fasted animals following oral administration. In dogs, ~40% of the drug is excreted in urine unchanged with excretion of unchanged drug in feces being the other main route of drug elimination; approximately 15% of the drug is metabolized by the liver in dogs. Marbofloxacin has low plasma protein being 9.1% in dogs and 7.3% in cats. In cats 70% of the oral dose is excreted in urine as marbofloxacin and metabolites with ~85% as unchanged drug. At oral doses of marbofloxacin in dogs (2.5 mg/kg, 5 mg/kg) the C_max_ (µg/mL) was 2.0 and 4.2 versus 4.8 in cats (5 mg/kg). The AUC values were 31.2, 64 and 70 respectively. Paradis et al. studied the clinical efficacy of marbofloxacin tablets (2.75 mg/kg once daily for 21 or 28 days) [227]. Of 72 dogs, 62 (86.1%) had superficial pyoderma and 10 (14%) had deep pyoderma with 39 dogs having a history of prior pyoderma. *Staphylococcus intermedius* (*pseudintermedius*) was the dominant pathogen for skin lesions from 47 cases. Treatment success was 86.1% (62/72) with improvement in an additional six (8.3%) dogs; failure was noted in four (5.6%) dogs. Noted adverse events (6/81 dogs) included listlessness, anorexia, vomiting, soft stool, flatulence and polydipsia.

For skin and skin structure infections, the recommended duration of therapy is 2–3 days beyond cessation of symptoms to a maximum of 30 days; for urinary tract infection therapy is recommended for at least 10 days.

Marbofloxacin pharmacokinetics in lactating cows was investigated after repeated intramuscular administrations and the pharmacodynamics against mastitis isolated strains was also studied. Schneider et al. (2004) reported for marbofloxacin an AUC of 6.45 and a C_max_ of 1.05 in milk [245]. Using an *E. coli* MIC_90_ value of 0.016, the AUC/MIC_90_ ratio was 107 and the C_max_/MIC_90_ ratio was 17; for *S. aureus* with an MIC_90_ of 0.23, these values respectively were 13 and 2. Pirro et al. (1997) suggested that MIC_90_ values for marbofloxacin against *E. coli* and *S. aureus* isolates were 0.06 and 0.5 µg/mL respectively [116]. Such values will clearly lower the AUC/MIC and C_max_/MIC ratios for marbofloxacin in breast milk. By comparison Fraatz and Krebber (2004) reported on enrofloxacin in milk where the AUC was 22.09 and the C_max_ 4.31 [84]. Based on an MIC_90_ of 0.06 against *E. coli*, the AUC/MIC_90_ was 368 and the C_max_/MIC_90_ was 72; such values for ciprofloxacin (MIC_90_ 0.03 µg/mL) were 736 and 144 respectively. For *S. aureus* (MIC_90_ 0.25) and enrofloxacin, AUC/MIC_90_ and C_max_/MIC_90_ values were 88 and 17 respectively and for ciprofloxacin (MIC_90_ 0.5) 44 and 9 respectively. The above noted data suggests enrofloxacin (with ciprofloxacin) has more favorable PK/PD values in milk than does marbofloxacin. Unpublished data (Elanco, data on file) suggests that when one considers both enrofloxacin and ciprofloxacin in plasma, the enrofloxacin/ciprofloxacin ratio for determining AUC is 55:45 and for C_max_ it is 70:30 based on the 5 mg/kg subcutaneous dose. For the 2.5 mg/kg subcutaneous dose, plasma AUC and C_max_ is 50:50 for enrofloxacin and ciprofloxacin. Clearly, even with AUC and C_max_ values set, the AUC/MIC and C_max_/MIC ratios can vary considerably based on the MIC value being used. As well, results may also vary considerably depending on if an MIC value is used based on a single isolate versus an MIC for 50%, 90% or 100% of strains tested.

The pharmacokinetics of intravenous or intramuscular marbofloxacin (2 mg/kg), inoculated in healthy (n = 8) or *M. haemolytica* infected calves (n = 8) was investigated [246] with plasma drug concentrations determined by HPLC. Respectively, the C_max_ (1.4 versus 2.32) and AUC (12 versus 22.2) values were higher in diseased versus in healthy animals following intramuscular injection. MIC values for the four isolates tested ranged from 0.04 to 0.78 µg/mL (mean MIC = 0.1 µg/mL). The AUC _0–24_ values in healthy animals ranged from 15.4 to 300 compared to 28.5 to 555 in diseased cattle. The C_max_/MIC ratios in healthy cattle ranged from 1.8 to 35 versus 3 to 58 in diseased cattle respectively with similar values seen following subcutaneous administration.

Marbofloxacin is approved for treatment of bovine respiratory disease in cattle (>14 weeks of age) caused by *M. haemolytica* and *P. multocida*. The recommended dose is 10 mg/kg intramuscularly. Marbofloxacin is not for use in dairy cattle. It is not recommended for mass medication in cattle but rather for treating individual cases of BRD. From cattle, marbofloxacin is excreted unchanged in urine and feces. In clinical trials in Canadian feedlots, marbofloxacin was significantly (*p* < 0.05) more efficacious (92%) than the negative control group (52%) at 7 days post-treatment. The terminal plasma eliminates half-life (t_½_/h) was 10.7, 10.9 and 12.7 respectively.

Lapczak compared efficacy of marbofloxacin and ceftiofur in feedlot steers with naturally occurring BRD complex. Of 80 animals finished in a feedlot, 18 steers had clinical signs (mucopurulent nasal secretion, alternations to lung auscultation, leukocytosis and decreased intake of dry matter) of BRD [247]. Animals (three/group) were randomly assigned to receive either one dose of marbofloxacin (8 mg/kg Sc), two doses of marbofloxacin (8 mg/kg Sc—with a 24 h interval) or a single dose of ceftiofur (93.3 mg/kg IM) on day 0. Treated animals were monitored over 7 days. Marbofloxacin-treated animals with two doses showed absence of pneumonia on day 7 whereas those that received a single dose of marbofloxacin or ceftiofur showed signs of mild pneumonia (*p* = 0.01). Animals receiving two doses of marbofloxacin had high drug matter intake on days 2 and 3 as compared to the other regimens (*p* = 0.05). A higher incidence of pneumonia was seen in the single dose treatment groups (*p* = 0.02). the authors concluded that the two-dose marbofloxacin treatment eliminated clinical signs of pneumonia and led to quicker dry matter uptake and return to productivity.

Lhermie and colleagues reported on marbofloxacin for BRD in young bulls from French farms [248]. A total of 195 bulls between 7 and 10 months of age and with an average body weight of 299 kg were enrolled. Clinical signs of BRD included moderate/severe signs of depression, nasal discharge, cough, increased respiratory effort and rectal temperature ≥39.7 °C. Animals were divided into two groups—early adjusted dose or late standard dose based on time of detection of BRD. Based on clinical observations and increasing temperature, the early treatment group received 2 mg/kg of marbofloxacin; the late treatment group received 10 mg/kg of marbofloxacin. Follow up was for 30 days. First and relapse treatments were calculated as was efficacy on day 10. Relapse treatment (florfenicol or tulathromycin) were given as needed. Some animals had received prophylaxis (tildipirosin 4 mg/kg BW) while other did not. Prophylaxis was administered at the auction market place the day before entering farms. In herds without prophylaxis, mean proportions of first and relapse treatments respectively were 58 ± 14 and 18 ± 2% in the early treatment group; for the late treatment groups, those values respectively were 39 ± 26 and 2 ± 3%. The proportion of first line treatments was significantly higher (*p* < 0.05) in the early versus late treatment groups—regardless of prophylaxis treatment. In both groups, clinical scores and rectal temperature decreased significantly (*p* < 0.05) 24 h after initiation of treatment.

Thomas et al. conducted a multicenter controlled, randomized, blind trial comparing marbofloxacin (2 mg/kg 24 h for 4 days) to tilmicosin (10 mg/kg as a single SC injection) for BRD [249]. Animals were on farms in Belgium, Britain and France. Animals were examined clinically up to eight times over 28 days. Cure rates at day 4 were 84% for marbofloxacin and 82% for tilmicosin and not statistically different. The overall clinical scores were significantly lower after day 1 in the marbofloxacin treated group (*p* < 0.05) and there were no differences in relapse rates or average daily weight gain between groups. The authors concluded marbofloxacin had comparable but faster efficacy and better local injection site tolerance than did tilmicosin for BRD treatment.

### 11.5. Pradofloxacin

Pradofloxacin is an advanced generation fluoroquinolone antibacterial agent developed specifically for and approved for use in veterinary medicine—initially in companion animals and more recently in food animals. Pradofloxacin shows increased potency (lower MIC values) against clinically important Gram-positive pathogens than does other fluoroquinolone agents. Against susceptible strains of Gram-negative pathogens, pradofloxacin in vitro activity is similar or equivalent to other fluoroquinolones. By MPC testing, pradofloxacin had the lowest MPC values against canine strains of *E. coli* and S. pseudintermedius than did other quinolones. Pradofloxacin has a favorable PK/PD profile with a mean maximum serum drug concentration in dogs of 1.5 µg/mL (based on 3 mg/kg) and is widely distributed to various tissues—in some instances with drug concentrations in excess of serum concentrations. Pradofloxacin is a bactericidal drug. In clinical trials, pradofloxacin was found to be clinically equivalent to comparator drugs but had superior microbiological cure rates.

Drug distribution to various compartments/tissues allows for consideration of drug inhibiting/killing target pathogens at those specific anatomical locations. Renally excreted drugs have higher urine drug concentrations than those in serum or other compartments. Unfortunately, antimicrobial concentrations achievable and/or sustainable in many compartments (normal or inflamed) are unknown for many antimicrobial agents depending on the anatomical location. As such, determining optimal versus adequate versus inadequate therapy is challenging. This has become especially critical in today’s environment where suboptimal or inadequate therapy is thought to precipitate antimicrobial resistance.

From in vitro measurements with pradofloxacin against key veterinary pathogens, MPC values were found to be low for *S. pseudintermedius* strains and *E. coli* strains at 0.125 ug/mL. These values are well below the reported drug concentration values for pradofloxacin in skin (4.5 µg/mL), urine (3.4 µg/mL) or serum (1.2–1.5 µg/mL). When considering PK/PD parameters, C_max_/MIC and AUC/MIC ratios are very favorable for clinical cure and bacteriological cure. Such values suggest a low propensity for pradofloxacin to select for drug-resistant subpopulations given that the skin drug concentrations exceed the MPC values by 36 fold and in urine by 27 fold.

From recently published/presented studies, pradofloxacin was found to rapidly kill bacteria when canine isolates of *S. pseudintermedius* and *E.coli* were exposed to the drug. Comparator agents were cefazolin, cefovecin and doxycycline. Kill experiments were run as either 3 h or 24 h experiments. Kill experiments were conducted using clinically relevant drug concentrations: MIC, MPC, MSDC and MTDC.

Bacterial densities tested ranged from 10^5^ (100,000) colony forming units (CFU)/milliliter to 10^9^ (1 billion) CFU/mL. This range of bacterial densities covers low and high bacterial burdens that may be seen during infection. From published investigations, statistically significant differences were seen in favor of pradofloxacin versus the comparator agents for killing of the bacterial strains tested as well as the speed in which the organisms were killed and over the range of the bacterial densities tested [154,250]. For example, at the MTDC killing of *S. pseudintermedius* strains by pradofloxacin was statistically superior (*p* value—0.5–0.001) to comparator drugs (cefovecin, cefazolin, doxycycline) following 10 min of drug exposure. Statistically significant differences were seen for pradofloxacin versus comparators at multiple time points, varying drug concentrations and over a range of bacterial densities tested.

In vitro data shows pradofloxacin to be bactericidal and rapidly kills key companion animal pathogens. Such observations impact bacteriological cure and clinical cure and reduce the likelihood for relapse and resistance selection.

Bacteriological cure is a prerequisite of clinical cure. Clinical cure in the absence of bacteria eradication may lead to persistence or relapse of infection.

MIC and MPC measurements have been completed with pradofloxacin against key companion animal pathogens such as *E. coli* and *Staphylococcus pseudintermedius* and *Staphylococcus aureus*. Wetzstein et al. [25] compared MIC values for pradofloxacin and other quinolones against a standard laboratory strain of *E. coli* (American Type Culture Collection (ATCC) strain #8739) and reported the following values; pradofloxacin 0.015–0.03 µg/mL, enrofloxacin 0.03–0.06 µg/mL, marbofloxacin 0.03 µg/mL, danofloxacin 0.06 µg/mL and orbifloxacin 0.125 µg/mL. By comparison, MPC values were 0.2–0.25 µg/mL, 0.3–0.35 µg/mL, 0.25–0.3 µg/mL, 0.5–0.55 µg/mL and 1–1.25 µg/mL. While pradofloxacin has the lowest MPC values of the compounds summarized, other compounds such as enrofloxacin and marbofloxacin also had low MPC values that would be within therapeutic drug concentrations when considering drug pharmacology. In contrast, the differences were more striking when MIC and MPC values were determined for the aforementioned compounds tested against *S. aureus* ATCC 6538. MIC values for pradofloxacin were 0.03–0.06 µg/mL as compared to 0.06–0.125 µg/mL for enrofloxacin, 0.25–0.5 µg/mL for marbofloxacin, 0.125–0.25 µg/mL for danofloxacin and 0.5 µg/mL for orbifloxacin. A greater difference was seen for the measured MPC values; 0.5–0.6 µg/mL for pradofloxacin, 3–3.5 µg/mL for enrofloxacin, 3–3.5 µg/mL of marbofloxacin, 10–11 µg/mL for danofloxacin and 8–9 µg/mL for orbifloxacin. As such, pradofloxacin had substantially lower MPC values than the other quinolones tested. Wetzstein et al. also went on to test pradofloxacin and other quinolones against other strains of *E. coli*, *S. aureus* and *S. pseudintermedius* and found similar results—i.e., lowest MPC results for pradofloxacin.

The in vitro activity of pradofloxacin against key anaerobic bacteria was determined. Silley et al. (2007) reported MIC_90_ values for pradofloxacin, enrofloxacin, difloxacin, ibafloxacin and marbofloxacin against several anaerobic genus of bacteria [166]. For *Clostridium* species (spp.), the MIC_90_ value for pradofloxacin was 0.5 µg/mL as compared to 2–8 µg/mL for the other agents tested. Against *Bacteroides* spp., *Fusobacterium* spp. and *Prevotella* spp., MIC_90_ values for pradofloxacin were 1 µg/mL as compared to 4–32 µg/mL, 16–64 µg/mL and 4–16 µg/mL respectively for the other agents tested. Against *Porphyromonas* spp., *Sporomusa* spp. and *Propionibacterium* spp., MIC_50_ values were 0.062–0.25 µg/mL for pradofloxacin and these values were lower than for the other agents tested. When all strains (n = 141) were considered together, no strain had an MIC to pradofloxacin >2 µg/mL and the MIC_90_ value was 1 µg/mL. The MIC_90_ values for the other agents tested against all strains were as follows: enrofloxacin 16 µg/mL, difloxacin 16 µg/mL, ibafloxacin 16 µg/mL and marbofloxacin 8 µg/mL. For 310 strains of *Porphyromonas* spp. tested against pradofloxacin and metronidazole, MIC_90_ values were 0.125 µg/mL and 0.25 µg/mL respectively; against 320 strains of *Prevotella* spp., MIC_90_ values were 0.25 µg/mL and 0.5 µg/mL respectively. The *Porphyromonas* spp. and *Prevotella* spp. isolates were collected from canine clinical cases from six European countries [123]. To date, published MPC values have not been determined for pradofloxacin or other veterinary quinolones against anaerobic organisms.

Considering drug pharmacology of pradofloxacin in dogs and cats, the C_max_ drug concentration is ~1.45 µg/mL in dogs as compared to ~2.1 µg/mL in cats. The AUC is ~12.9 in dogs and ~8.4 in cats. As previously mentioned, MIC_90_ values for pradofloxacin against *E. coli* and *S. pseudintermedius* were reported to be 0.016 µg/mL and 0.063 µg/mL respectively; MPC_90_ values were 0.125 µg/mL and 0.125 µg/mL. The C_max_/MIC ratio in dogs for *E. coli* and *S. pseudintermedius* are 90.6 and 23.0; in cats 131.3 and 33.3 respectively. The C_max_/MPC ratio in dogs for *E. coli* and *S. pseudintermedius* are 11.6 and 11.6; for cats 16.8 and 16.8. For dogs, the AUC/MIC for *E. coli* and *S. pseudintermedius* are 806.3 and 204.8; in cats 525 and 133.3 respectively. The AUC/MPC ratios in dogs for *E. coli* and *S. pseudintermedius* were 103.2 and 103.2; in cats 67.2 and 67.2 respectively.

Bacterial urinary tract infections are rare in cats [251]. Litster et al. determined the clinical efficacy and palatability of pradofloxacin oral suspension (2.5%) in cats with lower urinary tract infections [232]. Seventy-eight cats with culture positive lower UTI were allocated to three groups: pradofloxacin (n = 27), doxycycline (n = 23) or amoxicillin/ clavulanate (n = 28). There were no differences in urinalysis characteristics between cats in each treatment group. Treatment durations were as follows: pradofloxacin median 18 days (range 11–30 days), doxycycline median 28 days (range 14–39 days) and amoxicillin/clavulanate median 21 days (range 11–46 days). Treatment duration was statistically shorter for pradofloxacin (*p* < 0.05) than for the other two drugs. All cats in the pradofloxacin treatment group had negative cultures post-treatment compared to three positives in each of the other two groups. Interestingly, the same organisms isolated pre-treatment were recovered post-treatment and all strains were considered susceptible to the treatment antibiotic. All agents were considered palatable by owner questions. Safety concerns were not identified. It was concluded the pradofloxacin 2.5% oral suspension was highly effective for treatment of bacterial lower UTI in cats.

*Mycoplasma hemofelis* is the cause of infectious anemia in cats that can lead to death. Dowers et al. reported on pradofloxacin treatment for experimentally induced *M. hemofelis* infection in cats [252]. A total of 23 pathogen free cats (13 castrated males, 13 intact females) were assigned to one of four groups: 1) doxycycline—5 mg/kg PO q24h, 2) low dose pradofloxacin—5 mg/kg PO q24h, 3) high dose pradofloxacin—10 mg/kg PO q24h and 4) untreated. Twenty-five days before start of study, two donor cats were inoculated with 1 mL intravenously with blood from a cat chronically infected with *M. hemofelis*. Donor cats were also given 20 mg intramuscularly to maximize bacteremia. For the study cats, each cat was inoculated intravenously with blood from each of the two donor cats. Blood for inoculation was collected from the donors and mixed from both animals prior to inoculation of the study cats. Treatment duration was 6 weeks and blood was collected from each cat at multiple timed intervals over the study duration. Rectal temperature, respiration rate, mucus membrane, attitude (lethargy [depression]), appetite and need for fluids was recorded. At least one clinical sign (fever, icterus, pale mucous membranes, lethargy/depression, inappetence) was seen in all cats. All cats were PCR positive but with varying copy numbers. The low dose pradofloxacin group copy numbers were significantly lower than those of the doxycycline group on days 28, 32, 35 and 42 and for the high dose pradofloxacin group. Copy numbers were lower than the doxycycline group on days 25 and 28. All treatment groups had lower copy numbers than did the control group. Each of the six pradofloxacin treated cats had negative results from *M. hemofelis* DNA in blood after the treatment period. The authors concluded pradofloxacin may be more effective at long term *M. hemofelis* clearance.

Hartmann et al. determined the efficacy of pradofloxacin in cats with *Chlamydophila felis* or *Mycoplasma* associated respiratory tract disease [253]. A total of 39 cats had signs of upper respiratory tract infection. This was a placebo-controlled double blind clinical trial and cats were assigned to receive either pradofloxacin 5 mg/kg q24h oral or doxycycline 5 mg/kg q24h oral for 42 consecutive days. *C. felis* and Mycoplasma spp. was found in 23 and 20 cats respectively at the start of the study. Improved clinical signs were seen within the first week of therapy in both groups and DNA copy numbers declined rapidly. Mycoplasma spp. was completely eliminated in both groups; four cats treated with pradofloxacin remained PCR positive for *C. felis* whereas *C. felis* was eliminated from all cats receiving doxycycline. From this study it was concluded that both agents had good efficacy against the target pathogens with improvement in clinical signs; however, pradofloxacin may not have eliminated infection in all cats.

Mueller and Stephan in a multi-centered, blinded, randomized parallel trial investigated pradofloxacin for the treatment of canine deep pyoderma [230]. A total of 158 dogs were randomized to two treatment groups: pradofloxacin (n = 80) 3 mg/kg BW OD oral or amoxicillin/clavulanate acid (n = 78) 12.5 mg/kg BW bid. Dogs were evaluated weekly for 3 weeks and every 2 weeks thereafter. Treatment ranged from 14 to 63 days with follow up examination after 14 days post-treatment for record relapses. Maximal treatment period was 9 weeks with maximal evaluation at 11 weeks. The predominant bacterial pathogens included *S. pseudintermedius* (44.7%), *Staphylococcus* spp. (17.1%), *Pseudomonas* spp. (7.9%), *E. coli* (5.2%) and *Proteus* spp. (3.5%). A diagnosis of pyoderma was based on clinical presentation and a positive bacterial culture.

Stephan and colleagues investigated pradofloxacin for canine pyoderma and wound infections under field conditions. The overall clinical care rate was 86.4% for pradofloxacin treated animals versus 81.6% for those given amoxicillin/clavulanic acid [254]. Of 56 dogs receiving pradofloxacin 48 dogs achieved clinical remission, four dogs improved and four dogs did not respond. Recurrence was not seen in any dogs after 11 weeks. For 51 dogs receiving amoxicillin/clavulanic acid, thirty-seven dogs had clinical remission, three dogs showed clinical improvement, five dogs showed no response and for six dogs recurrence of clinical signs appeared within 2 weeks of end of therapy (*p* = 0.0082 compared to pradofloxacin). The authors concluded pradofloxacin was efficacious and comparable to amoxicillin/clavulanic acid for deep canine pyoderma. Reduction in diseased skin surface was significantly higher (*p* = 0.0367) in pradofloxacin treated animals.

Canine wound infections were investigated in a controlled, blinded, randomized multi-center clinical field trial (Elanco data on file). The study included dogs of varying breeds, ages and body weights. Wound swabs were collected on day 0 and a wound score was assigned. Dogs were re-evaluated on days 7 and 14 and efficacy determined by comparing wound scores following therapy to those at day 0. Of 137 dogs, 67 received pradofloxacin tablets 3 mg/kg BW and were compared to 70 dogs that received amoxicillin/clavulanic acid 12 mg/kg bid for 7 days. From wounds, predominant bacterial species from some cases included *S. pseudintermedius* (*intermedius*) (46.8%), *Streptococcus* spp. (15.6%), *E. coli* (9.6%), *Pseudomonas* spp. (2.8%) and *Proteus* spp. (2.3%). Pradofloxacin was non-inferior to amoxicillin/clavulanic acid; however, pradofloxacin showed a trend to wound statistical superiority on day 14 (*p* = 0.0502).

Pradofloxacin was compared to placebo for clinical effectiveness for skin injection in cats (Elanco data on file). The study was a multi-center (16 sites) field study that was masked and randomized. Cats received either pradofloxacin 7.5 mg/kg or 0.3 mL/kg of placebo (vehicle without active ingredient) for 7 consecutive days. Of 1/6 animals receiving pradofloxacin, the percentage of cats cured was 73.4% as compared to 38.9% (*p* = 0.0053) of 66 animals receiving placebo.

Pradofloxacin oral suspension (5 mg/kg BW) was evaluated in cats for treatment of wound infection and abscesses and compared to amoxicillin/clavulanate (12.5 mg/kg bid) (Elanco data on file). A total of 156 cats were studied—74 treated with pradofloxacin and 82 with amoxicillin/clavulanic acid. In the pradofloxacin group, thirty-one cats had wound infection, thirty-four had abscesses and nine had both wound and abscesses: thirty-four, forty-two and six respectively in the amoxicillin/clavulanic acid group. Predominant bacterial species isolated included *Pasteurella* spp. (24.33%), *Staphylococcus* spp. (18.5%), *S. intermedius* (*pseudintermedius*) 15.3%, *Streptococcus* spp. (12.6%), *E. coli* (7.7%) and *Pseudomonas* spp. (5.4%). Cure rates at end of study were 97% for pradofloxacin and 99% for amoxicillin/clavulanic acid treated cats. Significant differences in wound and general condition scores were not different between groups.

Pradofloxacin (3 mg/kg) was evaluated for treatment of urinary tract infections in dogs in a controlled, randomized, blinded multi-center study (Elanco data on file). Comparator treatment was amoxicillin/clavulanic acid (12.5 mg/kg). Dogs with signs of upper or lower tract infection or prostatitis were included. Treatment duration was 7–21 days. Urine specimens for culture and susceptibility were collected prior to initiation of therapy and 7 days after the end of therapy (by catheterization/cystocentesis). A total of 85 animals treated with pradofloxacin and 77 with amoxicillin/clavulanic acid completed the study and clinical rates were 89% and 84% respectively. The predominant bacteria isolated were *E. coli* (44.6%), other *Enterobacteriaceae* (15.7%), *Staphylococcus pseudintermedius* (9.6%), *Proteus mirabilis* (7.2%), *Pseudomonas* spp. (7.2%) and *Klebsiella* spp. (3.0%). A total of 52% and 56% of dogs treated with pradofloxacin and amoxicillin/clavulanic acid had pre-treatment positive urine for bacteria. The overall bacteriological cure rate was significantly greater (*p* = 0.002) in the pradofloxacin treated group (85.3% versus 48.0% in amoxicillin/clavulanic acid group). For dogs with cystitis, bacteriological cure rates were 88.5% in the pradofloxacin group versus 52.4% in the amoxicillin/clavulanic acid group; for prostatitis bacteriological cure rates were 75% and 50% respectively.

Periodontal disease is a common problem in dogs over the age of 3 years. In veterinary primary care and according to Wallis and Holcombe, visual oral assessment suggests a disease frequency of 9.3–18.2% [255]. However, detailed examination of dogs under anaesthesia indicated a prevalence ranging from 44 to 100%. From a primary care veterinary setting in the United Kingdom, O’Neill and colleagues reported a one-year prevalence of 12.52% periodontal disease based on a random sampling of 22, 333 dogs [256]. Pradofloxacin was evaluated for efficacy as adjunctive therapy to mechanical or surgical treatment in dogs with severe gingival or periodontal tissue disease. Dental procedures in dogs can induce bacteremia [257,258]. Nieves et al. reported from a study of 20 adult greyhound dogs that all dogs studied had bacteremia within 40 min from the initiation of dental procedures. Bacteria isolated from blood was the same genera isolated from plaque. Antimicrobials given pre- and post-operatively reduces risk for bacteremia-associated disease and has been recommended [259]. Weese et al. reported on antimicrobial use in dogs and cats undergoing dental procedures in the USA [260]. Data was from 713,901 dogs and 104,249 cats. Local or systemic antimicrobial agents were used in 16.4% of dogs and 14% in cats.

Pradofloxacin (3 mg/kg) was compared to clindamycin (5.5 mg/kg) for alleviation of periodontal disease associated clinical symptoms in a randomized multi-center controlled trial [261]. Some 125 male and female dogs were enrolled—64 pradofloxacin and 61 clindamycin. Treatment was once daily for seven consecutive days. The disease parameters determined under anaesthesia included pocket depth, bleeding on probing—both of which were improved in both treatment groups and not significant between different treatments. Pradofloxacin has reported activity against anaerobic bacteria [166]. Pradofloxacin therapy resulted in a significant reduction in the total anaerobic bacteria count (80%) from day 0 and day 13 but a similar change was not seen with clindamycin (8%).

Pradofloxacin (3 mg/kg OD) was compared to spiramycin/metronidazole (12.5 mg/75,000 IU/kg bid) (Elanco data on file) in dogs with periodontal disease with eight dogs in each treatment group. The principal clinical parameter assessed was loss of attachment (apical migration of epithelial attachment of gingiva and periodontal ligament). Both antimicrobial therapies significantly reduced loss of attachment without differences between therapies; however, the reduction in pradofloxacin treated animals was 0.47 mm compared to 0.32 in the comparator groups. Subgingival bacterial counts were significantly reduced by both treatments with the following organisms studied: *Actinobacillus actinomycetemcomitans*, *Porphyromonas gingivalis*, *Prevotella intermedia*, *Eikenella corrodens*, *Caprocytophaga orhracea*, *Porphyromonas canoris*, *Porphyromonas cangingivalis*, *Porphyromonas cansulci*, *Fusobacterim nucleatum* and *Campylocbacter rectus*. Results were recorded on days 1, 7, 14 and 28. At study start, 59.4% versus 56.3% of sites were positive in the pradofloxacin versus spiramycin/metronidazole treatment groups; by day 7, 43.7% versus 56.3% respectively; by day 14, 68.8% versus 75% respectively; by day 28, 73.1% versus 68.8% respectively. The impact of both treatments on Gram-positive to Gram-negative bacteria ratios was measured over 28 days. The proportion of Gram-negative bacteria at day 7 and 28 in the spiramycin/metronidazole group were 47.5 and 52.8% (71% at start of study) as compared to 23.6% and 25% (69.1% at start of study) in the pradofloxacin group. While both treatments reduced subgingival Gram-negative bacteria, the reduction in the pradofloxacin treated dogs was significantly lower at both day 7 and day 28 (*p* < 0.001).

Pradofloxacin (5 mg/kg OD for 5 days) was compared to amoxicillin/clavulanic acid (12.5 mg/kg bid for 5 days) treatment of upper respiratory tract infection in cats in a controlled, randomized, blinded multi-center field study [262]. A total of 80 cats received pradofloxacin and 68 cats treated with amoxicillin/clavulanic acid. The predominant bacteria (oropharyngeal swabs) were *Pasteurella* spp. (26.3%), *E. coli* (18.2%), *Staphylococcus intermedius* (*pseudintermedius*) (9.1%), *Streptococcus* spp. (7.3%), *Pseudomonas* spp. (7.3%) and *Staphylococcus* (4.7%). At 5 days after the end of therapy (day 10), the clinical response was 92% in the pradofloxacin group (70% clinical cure) as compared to 88% in the amoxicillin/clavulanic acid group (69% clinical cure). Cats were re-sampled at the end of therapy and bacteriological cure determine for each treatment group. Pathogen elimination was significantly higher (*p* < 0.001) in pradofloxacin treated cats (91%) as compared to those receiving amoxicillin/clavulanic acid (81%).

In another controlled, randomized, blinded multi-center field study, pradofloxacin (3 mg/kg OD × 5 days) was compared to amoxicillin/clavulanic acid (12.5 mg bid × 5 days) in cats with upper respiratory tract infections (Elanco data on file). All animals were bacteriological positive (pharyngeal swabs) prior to initiation of therapy. Predominant bacterial species isolated included *Staphylococcus* spp. (21.6%), *E. coli* (13.9%), *S. intermedius* (*pseudintermedius*) (13.2%), *Pseudomonas* spp. (5.2%) and *Pasteurella* and *Streptococcus* spp. in limited cases. A total of 127 cats were included in the efficacy analysis—pradofloxacin (n = 65) and amoxicillin/clavulanic acid (n = 61). Clinical response versus clinical cure on day 10 was pradofloxacin 75% (cure 71%) and amoxicillin/clavulanic acid 70% (cure 67%) and statistically significant differences between treatment groups was not seen.

As pradofloxacin has recently been approved for treatment of food animals (cattle and swine) PK/PD measurements in cattle have been completed. Foster et al. investigated the pharmacokinetics of pradofloxacin, florfenicol and tulathromycin in steers experimentally infected with *M. haemolytica* [85]. A total of 24 Holstein and Holstein/Jersey cross steers (6–15 months of age) weighing 87–390 kg (mean 238.73 kg) and in good health were used and randomly assigned into groups of three and blinded such that only the study coordinator was aware of the treatment groups. Random numbers were used to assign the order in which the treatment groups were prefilled. Treatment groups were pradofloxacin 10 mg/kg subcutaneously in the neck, tulathromycin 2.5 mg/kg subcutaneously in the neck and florfenicol 2.2 mg/kg subcutaneously in the neck. For infection, a *M. haemolytica* strain was concentrated to a final density of 1 x 10^9^ CFU/mL. Infection was with nebulization with 5 mL of 1 × 10^9^ CFU/mL until the entire contents of the nebulizer reservoir being delivered. Animals were examined every 12 h after nebulization by a blinded observer until they met criteria for treatment and then every 24 h until euthanasia. Variables evaluated included rectal temperatures, depression score and respiratory score. Blood samples were collected via catheter in the jugular vein. An ultrafiltration probe was inserted subcutaneously above the withers and interstitial fluid collected. Pulmonary epithelial lining fluid was also collected. During necropsy, specimens were collected from the lungs for bacterial culture. Specifically, for pradofloxacin and considering geometric means for plasma, interstitial fluid and pulmonary epithelial lining fluid respectively: C_max_ was 3.40, 0.73, 0.81; AUC was 13.19, 6.39, 27.53; half-life was 4.79, 7.88, 24.94. The mean plasma protein binding was at 45.75%. In considering the *M. haemolytica* strain used with an MIC to pradofloxacin of 0.015 ug/mL the C_max_/MIC and AUC/MIC ratios in plasma would be 226.67 and 879.33; for interstitial fluid 48.67 and 426; for pulmonary epithelial lining fluid 54 and 1835.33. In considering a C_max_/MIC ratio of 8–12 or an AUC/MIC ratio >100 as being relevant for successful clinical outcome and resistance prevention, pradofloxacin drug concentrations in cattle would greatly exceed these values in plasma and pulmonary fluid drug concentrations based on the above noted study.

Pradofloxacin has been approved for use in cattle intended for slaughter including beef calves ≥2 months of age, growing beef steers/heifers and beef bulls. The drug is also for use in cattle intended for breeding and <1 year of age. The clinical indication is for bovine respiratory disease (BRD) caused by *M. haemolytica*, *P. multocida*, *H. somni* and *M. bovis*. A multicenter natural infection study from three geographical locations in the USA evaluated pradofloxacin in cattle with clinical signs of BRD (depression, elevated rectal temperature and/or respiratory scores (Freedom of Information Summary NADA 141–550)). A total of 630 calves (bull, steer, heifer) ≥4 months of age and weighing between 340 and 602 lbs from five study sites were (100–140 animals/site) used. Animals had received vaccines, anti-parasitics and implants as part of normal incoming process upon arrival. Animals were examined daily for clinical signs of BRD as previously described. The criteria for study enrollment were (1) depression score of ≥2 and rectal temperature ≥104.0 °F or (2) respiratory score ≥2 and rectal temperature ≥104.0 °F. Calves were randomized at each site 1:1 to receive either pradofloxacin or saline and study personnel were blinded to the treatments. A total of 315 animals received pradofloxacin (10 mg/kg BW) once and 315 received 0.05 mL/kg BW of saline once with either injection being subcutaneous. Animals were observed for signs of BRD from study day 3 to study day 10 and temperature and respiratory/depression scores recorded. Specimens for microbiological processing were collected from each animal pre-treatment (double guarded, deep nasopharyngeal swab) and from all treatment failures. Lung tissue was collected from mortalities (euthanized or found dead).

Animals found morbid or with severe respiratory distress were removed from the study, euthanized and considered treatment failures. Additionally, treatment failures on study days three through nine were based on the following: (1) depression score ≥2 and rectal temperature ≥104.0 °F or (2) respiratory score ≥2 and rectal temperature ≥104.0 °F whereas treatment success (day 10) was defined as (1) depression score ≤1 and (2) respiratory scores ≤1 and rectal temperature ≥104.0 °F. A total of six calves were removed from the study for non-BRD related reasons. A statistically significant difference (*p* = 0.0089) was seen in favor of pradofloxacin treated animals (49.7%) as compared to saline treated animals (25.6%). A total of 365 isolates of *M. haemolytica*, 248 *P. multocida*, 106 isolates of *H. somni* and 159 isolates of *M. bovis* were reported based on a single isolate from each enrolled calf.

Pradofloxacin (intramuscular injection) was compared to saline for treatment of swine respiratory disease caused by *A. pleuropneumoniae*, *B. bronchiseptica*, *G. parasuis*, *P. multocida*, *S. suis* and *M. hyopneumoniae*. The study animals were 1200 commercial crossbreed weaned barrows and gilts. The animals were 3.5–13 weeks of age with weights ranging from 5.9 to 86.0 lbs and were enrolled from 10 study sites. Pradofloxacin was administered at 7.5 mg/kg once to 60 animals and saline was administered at 0.017 mL/kg IM once to 600 animals. Clinical signs necessary for study enrollment were a respiratory score ≥2, a depression score ≥2 and rectal temperature ≥104.0 °F. All animals were evaluated on day 7 and then euthanized. Pleural swabs and duplicate lung tissue samples were collected at necropsy. *M. hyopneumoniae* was detected by culture and polymerase chain reaction. A total of 29 pigs were removed from the study due to non-SRD reasons leaving 584 pradofloxacin treated animals and 587 receiving saline. There was a statistical significant difference on study day 7 (*p* = 0.0274) in favor of pradofloxacin (45.2%) treated pigs as compared to those receiving saline (34.2%). Bacterial strains recovered from study pigs included 111 *B. bronchiseptica*, 93 *G. parasuis*, 212 *S. suis*, 99 *P. multocida*, 37 *M. hyopneumoniae*. *A. pleuropneumoniae* was not recovered from study animals. Pradofloxacin was concluded to be effective for treatment of SRD.

Pradofloxacin (7.5 mg/kg) was evaluated in an infection induced model in swine using *M. hyopneumoniae* (Elanco study #202011). The study population consisted of 72 healthy female and castrated male crossbreed pigs age between 8 and 10 weeks and weighing between 44.1 and 96.4 lbs. All pigs were serologically negative for *M. hyopneumoniae*. Pigs had an acclimation period. There were 12 pens with 9–11 pigs/pen. *M. hyopneumoniae* was inoculated (endotracheal and intranasal with a strain known to produce lung lesions) to candidate pigs for three consecutive days. When 5% of inoculated pigs were observed with coughing on a single day and 4/5 randomly selected pigs has lung lesions scores > 5%, the six pigs from each pen were randomized to receive either pradofloxacin (n = 3) or saline (n = 3) and the remaining pigs removed. Coughing, depression and respiratory scores were recorded once on study days -6, 0 and 10 and twice on study days 3–9 and all pigs were euthanized on study day 10 for evaluation of lung lesions. There was a highly statistically significant difference (*p* = 0.0002) in favor of pradofloxacin for the mean total lung lesion score versus saline treated pigs, 11.7% versus 33.1% respectively. The study concluded pradofloxacin 7.5 mg/kg administered intramuscularly decreased lung lesions associated with *M. hyopneumoniae* in swine.

### 11.6. Orbifloxacin

Orbifloxacin is a broad-spectrum antibacterial fluoroquinolone drug. The compound is slightly water soluble and solubility increases in acidic and alkaline environments. The drug is rapidly and near completely absorbed from the gastrointestinal tract following oral administration with ~100% bioavailability. Approximately 20% of the drug is protein bound. Following a 2.5 mg/kg oral dose, the maximum plasma concentration was approximately 2.3 µg/mL. The plasma elimination half-life is approximately 6 h. Urine drug concentrations are approximately 100 µg/mL for ~2 h dropping to ~40 µg/mL by 24 h. Orbifloxacin penetrates most fluids and tissues. Shimizu et al. reported a maximum urine concentration of 383 ± 171 µg/mL from six healthy dogs administered 5 mg/kg [263]. Approximately 40–50% of the oral dose was excreted unchanged in urine. In cats, orbifloxacin is 18% protein bound. For cats, the maximum plasma concentration is 2.4 µg/mL (2 h) following a 7.5 mg/kg dose. The plasma half-life is 8 h. Investigation in dogs between orbifloxacin oral suspension and tablets found them to be bioequivalent but the same was not seen in cats where oral suspension provided lower and variable plasma levels then did tablets. Orbifloxacin is approved in the USA in dogs for urinary tract infections and in cats for skin infections (wounds and abscesses). Scott and colleagues studied the efficacy of oral orbifloxacin in 23 dogs with superficial and/or deep staphylococcal pyoderma [229]. The duration of therapy varied averaging 29 days versus 72 days (range 25–150 days) for dogs with superficial versus deep infection respectively. The rate of relapse was 18% within 3 months. The authors concluded orbifloxacin was a safe, effective and convenient antibiotic for treating dogs with superficial and deep staphylococcal pyoderma.

The post antibiotic effect (PAE) defines a period of ongoing suppression of bacterial growth after complete removal of the antibiotic. Harada et al. measured the PAE for orbifloxacin against two strains each of *E. coli* and *P. aeruginosa* [264]. At twice the MIC, the PAEs ranged from 0.28 to 0.93 h (mean 0.29 h) for *E. coli* and 0.18 to 1.18 h (mean 0.37 h) for *P. aeruginosa*. Continual exposure to 0.1, 0.2 and 0.3, the MIC resulted in post-antibiotic sub-minimum inhibitory concentration average effects 0.55, 1.11 and 2.03 h respectively for *E. coli* and 1.04, 1.40 and 2.47 h respectively for *P. aeruginosa*.

Davis et al. reported on the pharmacokinetics of orbifloxacin (2.5 mg/kg) in six horses after oral or intravenous administration [82]. Following oral dosing, the C_max_ was 1.25 µg/mL, T½ was 3.42, AUC was 6.16 h. µg/mL with 20.64% protein binding and 68.35% bioavailability. Following IV administration the AUC was 9.04 h. µg/mL.

In goats, Marins et al. investigated the pharmacokinetics and milk penetration of orbifloxacin (2.5 mg/kg BW) in six clinically normal Marciano–Granadina female goats aged 5–6 years and weighing between 45.8 and 62.3 kgs. Drug administration was by intravenous, subcutaneous and intramuscular injections. Regardless of route of drug administration, mean plasma C_max_ values ranged from 1.66 to 1.85 mg/L. In milk the mean C_max_ values ranged from 1.56 to 1.77 mg/L and the mean AUC values ranged from 6.36 to 7.58 mg/h/L. The absolute bioavailability following subcutaneous or intramuscular administration was 108.96 and 105.01% respectively.

Biofilms are problematic for antimicrobial therapy. Shimizu and Haradon investigated orbifloxacin to determine the minimum biofilm concentration (MBECs) against canine uropathogens including *E. coli* (n = 100), *S. pseudintermedius* (n = 5), *P. aeruginosa* (n = 5), *K. pneumoniae* (n = 5) and *P. mirabilis* (n = 5) [265]. The Calgary biofilm method was used and involves biofilm formation on peg placed in wells of 96-well plates containing bacteria. Pegs with bacteria were then exposed to antimicrobial agents in doubling dilutions for 24, 72 and 168 h. For the 10 *E. coli* strains MIC values ranged from ≤0.03 to 0.125 µg/mL, ≤0.25–64 µg/mL and ≤0.25–2 µg/mL following 24, 72 and 168 h of drug exposure respectively; the corresponding MBEC values were 128–2058 µg/mL, ≤0.25–128 µg/mL and ≤0.25–2 µg/mL respectively. For *S. pseudintermedius* strains, MIC values ranged from 0.125 to 0.5 µg/mL and the MBEC values following 24, 72 and 168 h of drug exposure ranged from 64 to 2048 respectively. For *K. pneumoniae* and *P. mirabilis*, the MIC and MBEC values were similar to those seen for *E. coli* strains. The authors concluded that the drug exposure time and bacterial species impact the efficacy of orbifloxacin treatment of biofilm related UTIs in dogs. This study was an in vitro investigation and efficacy determination in dogs with biofilm associated UTIs was not done.

*Edwardsiellosis* is a potential significant bacteria pathogen in aquaculture. The principal species is *Edwardsiella tarda*. Ibrahem and colleagues reported on the bioavailability of orbifloxacin in African sharptooth cat fish (*Clarias gariepinus*) and its efficacy for controlled induced *Edwardsiellosis* at 7 days (early stage) and 15 days (late stage) post infection [266].

Male and female fish (n = 720 total) weighing 50 ± 2 g were used and were negative for *E. tarda*. Five groups of anesthetized experimentally infected (0–2 mls intraperitoneally of a 2.4 × 10^4^ cfu/mL inocula) fish (n = 40/group) were investigated as follows: (1) 50 mg-1 water/day starting at day 7 post (2) 50 mg-1 water infection 15 days post-treatment, (3) non-infected but exposed to orbifloxacin at same dose as groups 1, 2, (4) infected and not treated and (5) not infected and not treated. Muscle concentrations of orbifloxacin were 0.03 mg/g tissue, at 2 days post-treatment, 0.015 mg/g tissue at 7 days post-treatment and undetectable at 10 days post-treatment. The MIC of the *E. tarda* isolate to orbifloxacin was 0.016 µg/mL. Clinically, group 1 had complete recovery as determined by failure to recover *E. tarda* from liver, kidney or muscles from infected fish at 72 h post-treatment. For the group 2 fish, *E. tarda* was not recovered at 96 h post-treatment; however, treated fish showed unhealed skin lesions. The authors concluded orbifloxacin as an effective antibacterial drug for control of *E. tarda* infection in fish with early treatment more successful.

## 12. Antimicrobial Stewardship

Antimicrobial stewardship arose out of the need to preserve antibiotic effectiveness in an environment of increasing antimicrobial resistance [267]. In human medicine, where some fluoroquinolones are available in intravenous (IV) and oral formulations, step down therapy from IV to oral was a key strategy for antimicrobial stewardship programs. One can consider the principles of stewardship to incorporate the right drug, at the right dose for the right duration in the right patient. Under “One Health”, stewardship encompasses evidence-based use of antimicrobial agents to safeguard animal health, preserve antimicrobial efficacy and reduce the spread of drug-resistant organisms. Lloyd and Page reviewed antimicrobial stewardship in veterinary medicine [268]. In their article they reviewed the World Health Organization (WHO) action plan points on antimicrobial resistance: (1) improving awareness of antimicrobial resistance, (2) strengthen knowledge/evidence through surveillance/research, (3) infection prevention and control to reduce the incidence of infection, (4) optimize antimicrobials in human/veterinary medicine and 5) sustained investment for new medicine, diagnostic tools, vaccines and other interventions. The review by Lloyd and Page did not specifically highlight any drug or drug classes but was rather an overview of antimicrobial stewardship. Antimicrobial stewardship in veterinary medicine presents some unique challenges. Whereas in human/veterinary medicine antimicrobials administered orally, parenterally or topically, in veterinary medicine antimicrobials may also be administered in feed or immersion baths (aqua culture). As such, stewardship with specific drugs or drug classes may be influence by drug administration methods.

## 13. Fluoroquinolone Toxicity

Fluoroquinolones are associated with adverse events in animals and some events vary by species. For example, enrofloxacin (and related quinolones) at higher dosages can cause acute retinal degeneration that may result in blindness in cats. For dogs with myasthenia gravis, quinolones can facilitate generalized weakness. Growing and immature puppies and foals are a concern with fluoroquinolones due to the potential for erosive arthropathias—cartilage damage in weight bearing joints. More common side effects include gastrointestinal disturbances (nausea, decreased appetite, vomiting, diarrhea), neurological events (tremors, restlessness, anxiety, seizures) at high doses and photosensitivity.

In food animals, adverse events are generally not a concern in terms of toxicity to animals and this is likely due, in part, to limited short course therapy. The concerns with fluoroquinolones use in food animals is the potential selection of antimicrobial resistance in microorganisms (e.g., *Salmonella* spp., *Campylobacter* spp.) that may be subsequently transmitted to humans via the food chain and thereby reduce antimicrobial effectiveness for the affected antimicrobials or antimicrobial drug classes.

## 14. Conclusions

Fluoroquinolones are important and critical antibiotics in both human and veterinary medicine and judicious use is essential. This narrative broad review is intended to capture the expanse of data with those antimicrobials in both human and veterinary medicine but with a veterinary medicine focus.

PK/PD modeling provides a framework for determining the drug exposure necessary for bacterial eradication. Additionally, differences between antimicrobials within the same class can be defined. To date, most PK/PD modeling has been based on MIC measurements and clearly more studies investigating MPC values are needed. PK/PD modeling has inherent limitations that include biological, clinical and systemic variables. Dosing optimization is aided by PK/PD modeling.

In vitro measurements define the spectrum of antimicrobial drugs, potency as defined by MIC and MPC measurements, bactericidal versus bacteriostatic based on kill measurements and the potential to block mutant cell growth (MPC) at clinically relevant drug concentrations. In vitro measurements do not correlate 100% with clinical outcomes and cannot include the biological variables in the human or animal body. MPC measurements remain a technical challenge for routine testing in diagnostic laboratories.

Regardless, such measurements provide important immunomodulating characteristics of antimicrobials has been an area of interest and investigation. Exactly how this manifests clinically remains an area of consideration. Airola and colleagues reviewed the “pleiotropic” effects of antibiotics and their potential clinical utility in humans. The benefits of immunomodulating effects of some antibiotics may be the chronic conditions.

As the prevalence of antimicrobial resistance increases, the clinical utility of antimicrobial agents will decrease dramatically. Combination therapy for pathogens that are currently successfully treated with single agents may become more commonplace. Whether new agents will be developed quickly enough is problematic. In the past, favor was shown to narrow spectrum agents, minimum dosages shown to be efficacious in clinical trials, and long duration of therapy. The net result (either directly or indirectly) has been the emergence of resistance. More recently the trend has been to kill the pathogens quickly. To do this, one may need to consider the use of more potent agents, higher dosages, and hopefully shorter duration of therapy. The net result of this approach remains unknown. One problem is the general sense that mutant subpopulations need not be considered because the agents still work clinically. We have argued above that subpopulations cannot be ignored because as they grow, they increase the speed at which resistance develops.

We have proposed an anti-mutant approach that is based on studies with fluoroquinolones, and now other agents in which a concentration range is observed in which resistant mutants are enriched. Standard therapy regimens often place antimicrobial concentrations in that range (MSW). Dosing to exceed the range, i.e., to exceed the MPC, is possible for some compounds with some pathogens. All other situations would require combination therapy.

Previously and prior to an abundance of MPC measurements, Smith et al. stated that the MPC had been stretched beyond its limits. It was argued that due to the principal mechanisms of resistance, MPC studies do not apply to aminoglycoside, beta-lactam and macrolide agents. While it may not be feasible to measure MPC with every agent and bacterium and horizontal transfer of resistance factors may require MPC being redefined to require several mutations rather than two for growth, the concept of the MSW is independent of resistance mechanism. It provides a general framework for deciding how many agents are needed. The major unanswered question is how well the window boundaries determined in vitro correlate with those present in vivo, since that relationship dictates how predictive agar plate studies will be.

Fluoroquinolones are broad spectrum, bactericidal and potent antimicrobial drugs that are clinically efficacious for number clinical indication. Judicious use is essential.

## Figures and Tables

**Table 1:** Current approved fluoroquinolones in veterinary medicine and indication in food animals.

Drug	Country	Organisms on Label	Clinical Indications
Danofloxacin [5]	USA Canada	*Mannheimia haemolytica* *Pasteurella multocida*	Treatment/control respiratory disease in cattle.
Enrofloxacin [6]	USA Canada	*Mannheimia haemolytica* *Pasteurella multocida* *Histophilus somni* *Mycoplasma bovis*	Treatment/control respiratory disease in cattle.
	USA Canada	*Actinobacillus pleuropneumoniae* *Pasteurella multocida* *Haemophilus parasuis* *Streptococcus suis* *Bordetella bronchiseptica* *Mycoplasma hyopneumoniae* *Escherichia coli* Colibacillosis—Swine	Treatment/control respiratory disease in swine.
Marbofloxacin [5]	Canada	*Mannheimia haemolytica* *Pasteurella multocida*	Treatment of respiratory disease in cattle.
Pradofloxacin [6]	USA (bovine)	*Mannheimia haemolytica* *Pasteurella multocida* *Histophilus somni* *Mycoplasma bovis*	Bovine respiratory disease.
	USA (swine)	*Bordetella bronchiseptica* *Glasserella* (*Haemophilus*) *parasuis* *Pasteurella multocida* *Streptococcus suis* *Mycoplasma hyopneumoniae*	Swine respiratory disease.

**Table 2:** Current approved fluroquinolones in veterinary medicine and indications in companion animals.

Drug	Country	Organisms on Label	Clinical Indications
Marbofloxacin [5]	USA		Skin and soft tissue infections. Urinary tract infections.
	Europe		Dogs Skin and soft tissue infections. Urinary tract infections. Respiratory tract infections.
Orbifloxacin [7]	USA	*Staphylococcus pseudintermedius* *Proteus mirabilis* *Escherichia coli* *Enterococcus faecalis*	Urinary tract infections—dogs.
		***Staphylococcus pseudintermedius*** *Staphylococcus aureus* Coagulase-positive *Staphylococcus* *Pasteurella multocida* *Proteus mirabilis* *Pseudomonas* spp. *Streptococcus equisimilis* *Klebsiella pneumoniae* *Escherichia coli* *Enterobacter* spp. *Citrobacter* spp. *Enterococcus faecalis* Beta-haemolytica *Streptococcus*	Skin and soft tissue skin infections—dogs.
	Europe	*Escherichia coli* *Proteus mirabilis*	Urinary tract infections—dogs. Skin and soft tissue infections.
Pradofloxacin [6]	USA	*Pasteurella multocida* *Streptococcus canis* *Staphylococcus aureus* *Staphylococcus felis* ***Staphylococcus pseudintermedius***	Skin infections in cats.
	Europe	*Staphylococcus pseudintermedius* *Escherichia coli* *Porphyromonas* spp. *Prevotella* spp.	Dogs Wound infections. Superficial and deep pyoderma. Acute urinary tract infections. Adjunctive therapy to mechanical or surgical periodontal therapy in the treatment of severe infections of the gingiva and periodontal tissues.
		*Pasteurella multocida* *Escherichia coli* ***Staphylococcus pseudintermedius***	Cats Skin and soft tissue infections. Acute infection of upper respiratory tract infections.
		*Staphylococcus aureus* *Escherichia coli* *Pasteurella multocida*	

**Table 3:** Pharmacokinetic variables for fluoroquinolones in various animals.

Drug	Dose	C_max_ (µg/mL)	T_max_ (h)	T_½_ (h)	AUC_0–24 (h)_ (µg/mL)	AUC_0-inf_ (µg/h/mL)	% Protein Binding	Bioavailability %
Danofloxacin								
Cattle [71]	6 mg/kg	1.25	3.2	4.8	9.4		36.4	92
Turkey [72]	7 mg/kg oral	1.17	2.13	9.74	7.70	8.95		78.37
Difloxacin								
Dog [73]	5 mg/kg	1.8	2.8	9.3		14.5	50.0	>95
Enrofloxacin								
Cats [74]	5 mg/kg	2.4	0.83	8.9		29.4	---	100
Dogs [74]	5 mg/kg	1.7	1	3.2		62.9	<30	95
Cattle [75]	2.5 mg/kg SC	0.2	1.7	---		1.4	46	
	8 mg/kg SC	0.8	2	7.3		7.5	46	
	12.5 mg/kg SC	0.96	4.8	6.8	8.7	14.9	46	
Donkeys [76]	7.5 mg/kg Intragastric	2.56	0.55	11.4		10.3		
Marbofloxacin								
Cats [5]	2.5 mg/lb	4.8	1.2	12.7		70	7.3	94
Dogs [5]	1.25 mg/lb	2.0	1.5	10.7		31.2	9.1	94
Dogs [5]	2.5 mg/lb	4.2	1.8	10.9		64	9.1	94
Pigs [77]	2.5 mg/kg	1.6	2	21.5		31.2		
Cattle [78]	10 mg/kg	7.9	1.3	17.5		52.7	30	>90
Orbifloxacin								
Cats [7]	Oral 7.5 mg/kg	3.01			7.57	33.98	18	
Dogs [79]	Oral 2.5 mg/kg	2.3	46 min	5.6				
	7.5 mg/kg	6.3	2	6.1		14.3	20	~100
Goats (plasma) [80]	2.5 mg/kg BW IV			4.12		6.15		~100
	2.5 mg/kg BW SC	1.85	1.25	4.99		6.47		
	2.5 mg/kg BW IM	1.66	0.87	3.34		5.98		
Goats (milk) [80]	2.5 mg/kg BW IV	1.56	1.83	1.84		6.36		
	2.5 mg/kg BW SC	1.73	2.00	1.93	9.04	7.58		
	2.5 mg/kg BW IM	1.77	2.00	1.94	6.16	7.43		
Goats [81]	2.5 mg/kg IV			8.63		12.65		
	2.5 mg/kg IM	1.76	1	17.77		19.66		155.5
Horse [82]	2.5 mg/kg IV			5.08	9.04		20.64	
	2.5 mg/kg oral	1.25	1.21	3.42	6.16			68.35
Pradofloxacin						60.8	20	~100
Cats [83,84]	3 mg/kg	1.2	0.5	9.8	4.9	5.9	30	>70
Cats [74]	5 mg/kg	2.1	0.5	9.3	8.3	9.3	30	>70
Dogs [83,84]	3 mg/kg	1.26	2.1	6.6	11.1	12.8	36	>95
Cattle	10 mg/kg BW SC	1.9	1	2.8		10.5		
Cattle [85]	10 mg/kg BW IM	3.4	0.74	4.79	13.19		45	
Pigs [86]	7.5 mg/kg BW IM	2.5	0.75	8.2		25.9		

**Table 4:** A summary of select clinical trials for veterinary-approved fluoroquinolones.

Drug	Treatment Route/Dose	Species	Condition	No.	Duration of Therapy	Clinical Outcome	Adverse Events	Ref.
Danofloxacin	6 mg/kg injectable	Cattle	BRD	128	One or two injections on successive days. One injection or a second injection on day 2 in animals with temperature ≥ 39.6 °C, moderate/severe signs of abnormal respiration or depression.	- Day 1—Rapid reduction in mean rectal temperature. - Rapid reduction in respiration and depression on days 4 and 10. - Day 4—66% breathing normally; 85% no signs of depression. - 54% of animal met criteria for dual dose.		[218]
	6 mg/kg	Cattle	Respiratory disease Enteritis Metritis Omphalitis	1019	One or tw0 injections on successive days.	Overall—94.8% response to therapy—84.1% cured. - Respiratory—79.8%. - Enteritis—92.6%. - Metritis—76.5% - Omphalitis—88.0%.		[219]
	6 mg/kg	Cattle	Diarrhea	267	Single SC injection or second injection on day 2 if required.	93.2–93.9% clinical improvement. - Significant reduction in severity of clinical signs on days 4 and 10 (p < 0.0001) and between days 4 and 10 (p < 0.05).		[220]
Enrofloxacin	18–20 mg/kg Oral Once daily	Dog	UTI	35	3 days.	Clinical cure 88.6%. Microbiological cure 77.1%.		[221]
	2.5 mg/kg Oral	Dog	Canine pyoderma	30	BID or 2–14 weeks.	28/30 (93.3%) excellent response at end of antibiotic therapy.		[222]
	10 mg/kg + 0.5% topical in an alginate gel TID	Dog	- Severe or very severe in dogs - Unsuccessfully treated with another antibiotic	55	- 8.03 ± 2.1 days severe. - 12.0 ± 2.4 for very severe.	32 severe cases—complete recovery. 23 very severe—complete recovery.	Inconsequential.	[223]
	5 mg/kg oral once daily	Dog	- Recurrent pyoderma	12	- 1–2 weeks beyond clinical recovery for recurrent vs. deep pyoderma respectively.	- Nine dogs superficial pyoderma. - Three dogs recurrent deep pyoderma. - Two dogs - 10/10 completed therapy—complete recovery.		[224]
	5% aqueous enro-C IM. Enrofloxacin—HCL-2 H2_0_	Dog	Leptospirosis	45	10 days followed by 6 days oral in gelatin capsules.	34 high risk. 11 medium risk. 82.2% negative after 3–5 days of therapy. 100% negative after 30 days.	Inconsequential.	[225]
Ibafloxacin	Oral 15 mg/kg	Dog	Superficial or deep pyoderma		41 ± 26 days.	- 1 week after therapy 74% response. - 1 month after therapy 70% cure/improved. - 3% relapse.		[226]
Marbofloxacin	Oral 2.75 mg/kg	Dog	Superficial pyoderma (n = 62) Deep pyoderma (n = 10)	72	21 or 28 days.	- Success 62/72. - Improved 6/72. - Failure 4/72.	AE in 4/72, listlessness, anorexia, vomiting, soft stool, flatulence, polydipsia.	[227]
	Oral 2.12 mg/kg	Dog	Severe and/or recurrent pyoderma lesions	39	10–21 days.	- 33/39 cure. - 1/39 improvement. - 1/39 small improvement. - 4/39 no response (11–60 days). - 15/39 relapse (3–191 days).	None reported.	
	Oral 2 mg/kg	Dog	Superficial or deep pyoderma		38 ± 21 days.	- 1 week after therapy 81% respond to therapy. - 1 month after therapy 70% cured/improved. - 11% relapse.		[226]
	Oral 2 mg/kg	Dog/Cats	Urinary tract infection	118/123	10 days UTI. ≤28 days prostatitis.	- 87.3% cure rate. - 96.0% bacteriological cure, no relapse.	None reported.	[228]
Orbifloxacin	Oral 2/5 mg/kg	Dog	Superficial and/or deep staphylococcal * pyoderma	23	- 21–40 days for superficial infection. - 25–150 days for deep infections.	- 95% resolution of infection. - 4/23 had recurrences within 3 months.	One case of presumed cutaneous drug reaction.	[229]
Pradofloxacin	Oral 3 mg/kg	Dogs (not approved for use in the US)	Deep pyoderma.	56	≤weeks.	- 48–56 clinical remission. - 45/56 improved. - 4/56 no response to therapy. - Clinical recurrence not seen.		[230]
	Oral 3 mg/kg	Dogs (not approved for use in the US)	Superficial/deep pyoderma	20				[231]
	Oral suspension 2.5%	Cats	Urinary tract infection.	27				[232]
	10 mg/kg BW. One dose SC in neck region.	Beef Cattle	BRD	90	One dose.	45:45—pradofloxacin vs. sterile saline. - Study day 7—71.1% vs. 25.6%. - Study day 10—60.0% vs. 20.9%. - Study day 14—44.4 vs. 16.3%.		**
	10 mg/kg BW. One dose SC	Cattle (bull, steer, heifer)	BRD	630	One dose.	315 pradofloxacin vs. 315 sterile saline. - Study day 10 49.7% vs. 25.6%.	None reported for any treatment.	***
	5 mg/kg q24	Cats	Feline rhinitis	13	Seven doses. Amoxicillin 22 mg/kg q12.	- 11/13 85% clinical resolution	Amoxicillin 22 mg/k q 12 for seven doses 10/15 (67%) clinical resolution.	[233]
	10 mg/kg q24	Cat	Feline rhinitis	12	Seven doses.	- 11/12 (92%) clinical resolution.		

** FOI NADA Study #201218. *** FOI Study 20251.

## Data Availability

The original contributions presented in this study are included in the article. Further inquiries can be directed to the corresponding author.
